# Maize 16-kD γ-zein forms very unusual disulfide-bonded polymers in the endoplasmic reticulum: implications for prolamin evolution

**DOI:** 10.1093/jxb/ery287

**Published:** 2018-08-02

**Authors:** Davide Mainieri, Claudia A Marrano, Bhakti Prinsi, Dario Maffi, Marc Tschofen, Luca Espen, Eva Stöger, Franco Faoro, Emanuela Pedrazzini, Alessandro Vitale

**Affiliations:** 1 Istituto di Biologia e Biotecnologia Agraria, CNR, Milano, Italy; 2 Dipartimento di Scienze Agrarie e Ambientali, Università degli Studi di Milano, Milano, Italy; 3 Department of Applied Genetics and Cell Biology, University of Natural Resources and Life Sciences, Vienna, Austria

**Keywords:** Cereal seeds, disulfide bonds, endoplasmic reticulum, genome-wide duplication, neofunctionalization, prolamins, protein bodies, protein evolution

## Abstract

In the lumen of the endoplasmic reticulum (ER), prolamin storage proteins of cereal seeds form very large, ordered heteropolymers termed protein bodies (PBs), which are insoluble unless treated with alcohol or reducing agents. In maize PBs, 16-kD γ-zein locates at the interface between a core of alcohol-soluble α-zeins and the outermost layer mainly composed of the reduced-soluble 27-kD γ-zein. 16-kD γ-zein originates from 27-kD γ-zein upon whole-genome duplication and is mainly characterized by deletions in the N-terminal domain that eliminate most Pro-rich repeats and part of the Cys residues involved in inter-chain bonds. 27-kD γ-zein also forms insoluble PBs when expressed in transgenic vegetative tissues. We show that in Arabidopsis leaves, 16-kD γ-zein assembles into disulfide-linked polymers that fail to efficiently become insoluble. Instead of forming PBs, these polymers accumulate as very unusual threads that markedly enlarge the ER lumen, resembling amyloid-like fibers. Domain-swapping between the two γ-zeins indicates that the N-terminal region of 16-kD γ-zein has a dominant effect in preventing full insolubilization. Therefore, a newly evolved prolamin has lost the ability to form homotypic PBs, and has acquired a new function in the assembly of natural, heteropolymeric PBs.

## Teaser

A prolamin paralog generated upon maize whole-genome duplication has changed its polymerization and solubility properties, allowing a new function in the assembly of maize protein bodies.

## Introduction

Prolamins are present only in the seeds of grasses, where they are usually the main proteins, and thus constitute the major global source of food protein (Shewry and Halford, 2002). Their most striking and unique cell biology feature is their accumulation within the lumen of the endoplasmic reticulum (ER) as very large heteropolymers, termed protein bodies (PBs; Shewry and Halford, 2002; Pedrazzini *et al.*, 2016). Most proteins that enter the ER are destined to be secreted or sorted to distal locations of the endomembrane system, whereas ER residents, which are mainly folding helpers, have specific amino acid signals that allow their retention/retrieval in the ER (Gomez-Navarro and Miller, 2016). Since these signals are not present in prolamins, the question arises as to what are the molecular features that determine prolamin ER residence and ordered PB formation.

Maize (*Zea mays*) prolamins are divided into four classes: α-zeins (>30 genes), γ-zeins (three genes), and δ-zeins and β-zeins (both single genes; Woo *et al.*, 2001; Xu and Messing, 2008). 27-kD γ-zein and β-zein are the oldest maize prolamins (Xu and Messing, 2008). Whole-genome duplications (WGD), particularly common in plants (Jiao *et al.*, 2011), are followed by rearrangements that can lead to gene loss or retention. In the latter case, functional buffering or neofunctionalization can occur, and play important roles in evolution (Chapman *et al.*, 2006; Kassahn *et al.*, 2009). About 5–12 million years ago, maize underwent WGD followed by allotetraploidization (Swigonˇová *et al.*, 2004). As a result, γ-zein, originally a single gene encoding a polypeptide of 27-kD and one of the most ancient maize prolamins, now has representatives in homologous regions of chromosome 7 (27- and 50-kD γ-zein; hereafter referred to as 27γz and 50γz) and chromosome 2 (16-kD γ-zein; 16γz). 16γz most probably originates from duplication of the 27γz gene followed by deletion events (Xu and Messing, 2008).

**Fig. 1.**
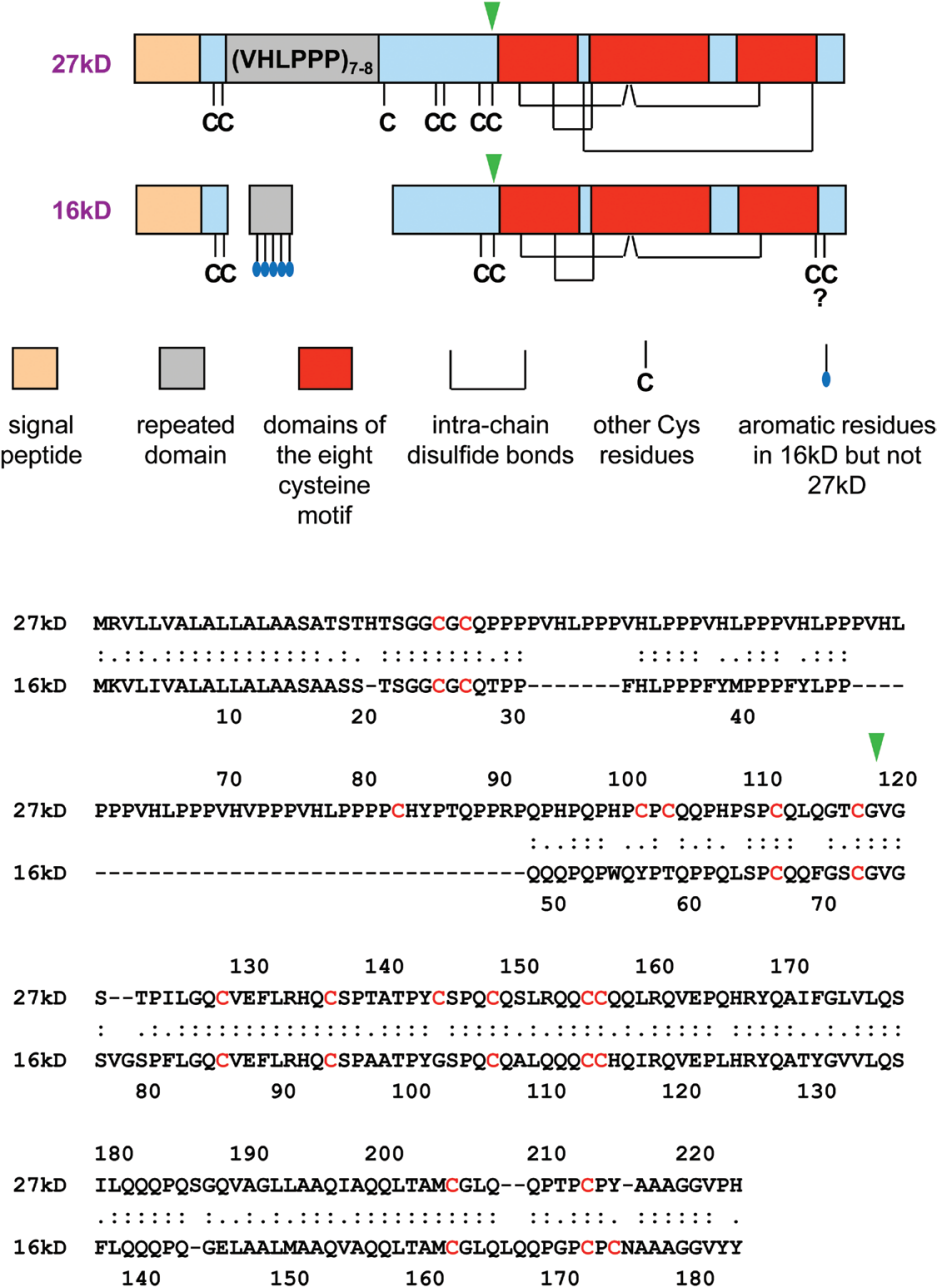
Schematic diagram and amino acid sequence of the 27-kD and 16-kD γ-zein primary translation products. The question mark indicates that the linkage status of the two Cys residues towards the C-terminus of the 16-kD γ-zein is not known. The green arrowheads indicate the points at which the N- and C-terminal regions of the two polypeptides were exchanged, to produce constructs 27/16 and 16/27. In the amino acid sequences, Cys residues are highlighted in red.

During endosperm development, γ- and β-zeins are synthesized first, forming a PB where α- and δ-zeins will later accumulate (Lending and Larkins, 1989). In the mature PB, β-zein, 27γz, and 50γz form the outer layer in contact with the luminal face of the ER membrane, whereas α- and δ-zeins form the inner core, with 16γz located at the interface between the core and the outer layer (Lending and Larkins, 1989; Yao *et al.*, 2016). Yeast two-hybrid data suggest that 16γz can interact with zeins of all classes (Kim *et al.*, 2002, 2006). 27γz expressed in vegetative tissues of transgenic plants forms homotypic PBs, indicating that no specific features of the maize endosperm ER are necessary to form a PB (Geli *et al.*, 1994). The primary sequence of 27γz (Fig. 1) consists of the transient signal peptide for translocation into the ER (co-translationally removed), followed by a region containing eight or seven (depending on the maize variety) repeats of the hexapeptide PPPVHL and seven Cys residues involved in inter-chain bonds that make the protein insoluble in non-reducing conditions, and finally a second region homologous to 2S albumins, which are vacuolar storage proteins present in various amounts in all land plants (Vitale *et al.*, 1982; Prat *et al.*, 1985; Mainieri *et al.*, 2014). 2S albumins belong to a larger class characterized by the eight-cysteine motif, consisting of four intra-chain disulfide bonds between three helical domains (Pedrazzini *et al.*, 2016; Fig. 1). This motif is also conserved in 27γz (Ems-McClung *et al.*, 2002). Progressive Cys-to-Ser mutation of the seven Cys residues of the N-terminal region lead to increased solubility and a parallel increase in the ability to leave the ER along the secretory pathway (Mainieri *et al.*, 2014). When the N-terminal region including the first six Cys residues is fused at the C-terminus of phaseolin, the vacuolar 7S storage globulin of common bean, the chimeric protein zeolin formed homotypic PBs in the ER (Mainieri *et al.*, 2004). Zeolin was instead efficiently secreted upon *in vivo* treatment with a reducing agent, or when its six Cys residues were mutated to Ser (Pompa and Vitale, 2006). Overall, these studies indicate that the N-terminal region of 27γz contains key information for PB assembly and that its Cys residues are necessary for this process.

16γz is mainly characterized by the loss of large part of the N-terminal, Pro-rich domain and three of its seven Cys residues (Prat *et al.*, 1987; Fig. 1). Additionally, its C-terminal region has lost one Cys residue of the eight-cysteine motif and has acquired a new one near the C-terminus, resulting in a new CysProCys sequence. This tripeptide could form an intra-chain disulfide bond (Yu *et al.*, 2012); however, it is not known whether this occurs in 16γz. The changes that have generated 16γz are noteworthy, since Cys residues are rarely lost once acquired during evolution (Wong *et al.*, 2011; Feyertag and Alvarez-Ponce, 2017). 16γz can thus provide information on the minimal requirements for PB biogenesis and the features that allow the formation of heteropolymeric maize PBs. Here, we show that, unlike 27γz, ectopically expressed 16γz remains in part soluble, mainly because of the mutations in the N-terminal region. 16γz is unable to form PBs, but it stably accumulates as polymers that markedly enlarge the ER lumen, giving rise to very unusual filamentous structures. These characteristics indicate neofunctionalization after WGD and cast light on the molecular basis for the specific organization of maize PBs.

## Materials and methods

### Analysis of maize PBs

Seeds from *Zea mays* inbred line W64A, collected at 25 d post-pollination and stored at –80 °C, were homogenized in a mortar using 5 ml g^−1^ ice-cold 100 mM Tris-Cl, pH 7.4, 1.0 mM EDTA (buffer H), 7% (w/w) sucrose, and cOmplete™ Protease Inhibitor Cocktail (Roche). After filtration through cheesecloth, the homogenate was loaded on two layers of 35% and 60% (w/w) sucrose in buffer H and centrifuged in a swinging rotor for 90 min at 4 °C, 78900 *g*_av_ (i.e. the average *g* calculated at the middle length of the tube). The 7% sucrose supernatant, the interface between 7% and 35% sucrose, and the interface between 35%, and 60% sucrose were collected. After denaturation in the presence of 1% SDS and 4% 2-mercaptoethanol (2-ME), proteins were analysed using 15% SDS-PAGE. As expected (Vitale *et al.*, 1982), zeins were at the interface between 35% and 60% sucrose, and hence this is termed the PB fraction. To treat PBs with different solvents, immediately after collection the PB fraction was first diluted with the same volume of buffer H and centrifuged for 10 min at 4 °C, 1500 *g*_av_. The PB pellet was then resuspended in one of the following solvents: (i) buffer H, 1% Triton X-100, 20 min, 4 °C; (ii) buffer H, 2 mM dithiothreitol (DTT), 20 min, 4 °C; (iii) buffer H, 4% 2-ME, 20 min, 4 °C; and (iv) 70% ethanol in H_2_O, 90 min, 25 °C. After each treatment, samples were centrifuged for 10 min at 4 °C, 1500 g, the pellet and supernatant were then denatured and analysed using 15% SDS-PAGE and staining with Coomassie Brilliant Blue. Protein Molecular Weight Markers (Fermentas, Vilnius, Lithuania) were used as molecular mass markers.

### Plasmid constructions

The pDHA vector containing the coding sequence of 27-kD γ-zein followed by the FLAG epitope DYKDDDDK (hereafter termed 27γzf) has been described previously (Mainieri *et al.*, 2014). To construct a similarly tagged 16-kD γ-zein (16γzf), a genomic fragment of *Z. mays* W64A comprising the coding and untranslated flanking sequences of 16-kD γ-zein (a kind gift from Angelo Viotti, CNR), identical to GenBank sequence EU953296.1, was amplified by PCR using the following oligonucleotides: 5’- ACTCAG*GTCGAC***ATG**AA GGTGCTGATCGTTGCCCTTG -3’ (where the SalI restriction site is in italics and the 16-kD γ-zein ATG start codon is in bold) and 5′- TCGATG*GCATGC*TCACTTGTCGTCGTCGTCCTTGTAGTCG TAGTAGACACCGCCGGCAGC -3′, (where the SphI restriction site is in italics and the reverse complement of the codons encoding the FLAG epitope is underlined). The sequence was restricted with SalI and SphI and reinserted into the similarly restricted pDHA vector for transient expression.

To produce transgenic Arabidopsis, the EcoRI fragments containing the 16γzf or 27γzf expression cassettes were excised from pDHA and subcloned into EcoRI-linearized pGreenII0179 (John Innes Centre, Norwich, UK). The *Agrobacterium tumefaciens* strain GV3101 containing the pSoup helper plasmid was transformed with the resulting constructs.

To prepare the chimeric construct 16/27, which is formed by the N-terminal primary sequence of 16γzf until Cys^73^ followed by the C-terminal sequence of 27γzf starting from Gly^118^ (see Fig. 1), DNA was synthesized (Integrated DNA Technologies, Leuven, Belgium) based on the two sequences and inserted into SalI/SphI restricted pDHA. To prepare the exactly reciprocal construct 27/16, the following DNA sequence was synthesized: from the Bpu10I restriction site of the sequence encoding 27γzf until its Gly^118^ codon (which corresponds to Gly^74^ of 16γzf), continuing with the 16γzf sequence from Val^75^ until its stop codon, and ending with a SphI restriction site. This sequence was used to substitute the Bpu10I/Sph1 fragment in pDHA encoding 27γzf.

### Transient expression in tobacco protoplasts

Transient expression was performed in protoplasts prepared from young (4–7 cm) leaves of tobacco (*Nicotiana tabacum* SR1) grown in axenic conditions, as described previously (Mainieri *et al.*, 2014). Resuspensions of 10^6^ protoplasts were transfected using 40 μg per million protoplasts of plasmid or, for co-transfections, 60 μg (25 μg of each plasmid plus empty pDHA to a final amount of 60 μg). After transfection and incubation for 20 h at 25 °C, protoplasts were either homogenized for protein blot analysis or subjected to pulse-chase labelling. Extraction of intracellular and secreted proteins in reducing or oxidizing conditions and protein blot analysis with rabbit anti-FLAG antibody (1:2000 dilution, Sigma-Aldrich) and the Super-Signal West Pico Chemiluminescent Substrate (Pierce Chemical, Rockford, IL) were performed as described previously (Mainieri *et al.*, 2014). Protein Molecular Weight Markers (Fermentas, Vilnius, Lithuania) were used as molecular mass markers.

Pulse-chase labelling was performed with 100 μCi ml^−1^ Easytag mixture of ^35^S-labelled Met and Cys (PerkinElmer) for 1 h at 25 °C. Chase was initiated by adding unlabelled Met and Cys to 10 mM and 5 mM, respectively. After incubation at 25 °C for the desired chase time, two volumes of ice-cold W5 buffer (Mainieri *et al.*, 2014) were added to each sample, which were then centrifuged at 60 *g*_av_ for 10 min. Collected protoplasts and supernatant (containing secreted proteins) were homogenized with two volumes of ice-cold 150 mM NaCl, 1.5 mM EDTA, 1.5% Triton X-100, 150 mM Tris-Cl pH 7.5, supplemented with cOmplete™ Protease Inhibitor Cocktail. After centrifugation at 10000 *g*_av_, the pellet was resuspended in the same buffer supplemented with 4% 2-ME and centrifuged again. The soluble fractions of the first and second centrifugation were immunoselected using the anti-FLAG antibody and protein A Sepharose (GE Healthcare) and analysed using SDS-PAGE and radiography, using ^14^C-methylated proteins (Sigma-Aldrich) as molecular mass markers. Radioactive proteins were detected using the Starion FLA-9000 Phosphoimage System (Fujifilm) and quantified using TotalLab Quant (TotalLab, Newcastle upon Tyne, UK).

### Expression in transgenic Arabidopsis

Transgenic *Arabidopsis thaliana* (ecotype Columbia) plants expressing 16γzf or 27γzf were produced by the floral dip method (Clough and Bent, 1998) with the transformed *A. tumefaciens* described above. Hygromycin-resistant T0 plants were identified and the homozygous progenies were selected. Experiments were then conducted using T2 or T3 plants. Plants were grown in soil at 23 °C under a 16/8 h light/dark cycle or in sterile conditions on half-concentrated Murashige and Skoog media (Duchefa Biochemie) supplemented with 10 g L^–1^ Sucrose and 0.8% (w/v) phyto agar (Duchefa Biochemie).

Leaves at 4–6 weeks old were homogenized in leaf homogenization buffer (150 mM Tris-Cl, pH 7.5, 150 mM NaCl, 1.5 mM EDTA, 1.5% Triton X-100, cOmplete™ Protease Inhibitor Cocktail), supplemented (reducing conditions) or not (oxidizing conditions) with 4% (v/v) 2-ME. Soluble and insoluble proteins were separated by centrifugation at 1500 *g*_av_ for 10 min at 4 °C. Samples were adjusted to 1.0% SDS, 4% 2-ME and analysed using SDS-PAGE followed by protein blotting with the anti-FLAG antibody (1:2000 dilution).

### Subcellular fractionation

Arabidopsis leaves at 4–6 weeks old were homogenized in 10 mM KCl, 2 mM MgCl2, 100 mM Tris-Cl, pH 7.8 (buffer A), and 12% (w/w) sucrose at 4 °C, followed by isopycnic ultracentrifugation using linear 16–65% (w/w) sucrose gradients in buffer A as described previously (Mainieri *et al.*, 2004). Fractions of 650 μl were collected; 40 μl samples of each fraction were denatured and analysed by SDS-PAGE, followed by protein blotting with anti-FLAG antibody or rabbit anti-endoplasmin serum (Klein *et al.*, 2006; 1:2500 dilution).

To determine the solubility of γ-zeins present in the different subcellular fractions, fractions around either 1.19 or 1.29 density were frozen to break membranes and were then pooled. An equal volume of buffer A was added and the suspension was centrifuged at 1500 *g*_av_ for 10 min, 4 °C. Supernatants (soluble proteins) were collected and denatured with SDS-PAGE denaturation buffer. Pellets were either resuspended in SDS-PAGE denaturation buffer or were further extracted with 70% ethanol in H_2_O for 90 min at 25 °C, and centrifuged at 1500 *g*_av_ for 10 min, 25 °C. The soluble fraction (ethanol-soluble) and insoluble pellet (ethanol-insoluble) were collected and denatured for SDS-PAGE.

### Velocity sucrose-gradient ultracentrifugation

Arabidopsis leaves were homogenized in ice-cold leaf homogenation buffer. The homogenate was loaded on top of a linear sucrose gradient (150 mM NaCl, 1 mM EDTA, 0.1% Triton X-100, 50 mM Tris-Cl, pH 7.5, 5–25% [w/v] sucrose). After centrifugation at 200000 *g*_av_ for 20 h, 4 °C, equal volumes of each fraction were analysed using SDS-PAGE and protein blotting. An identical gradient loaded with molecular mass markers was run in parallel. For velocity ultracentrifugation in reducing conditions, leaf homogenization buffer was supplemented with 4% 2-ME, and the sucrose gradient buffer was supplemented with 2% DTT.

### Electron microscopy

Tissue fragments (1–2 mm^2^) from fully expanded Arabidopsis leaves were fixed, embedded, and immunolabelled as previously described (Faoro *et al.*, 1991). Tissues were fixed in 1.2% glutaraldehyde and 3.3% paraformaldehyde in 0.1 M phosphate buffer, pH 7.4, at 4 °C for 2 h, post-fixed in 1% OsO_4_ in the same buffer for 2 h, dehydrated in an ethanol series, and then embedded in Spurr’s resin. For immunocytochemical localization, post-fixation was omitted and the embedding resin used was London Resin White. Immunolabelling was carried out on ultrathin sections mounted on nickel grids and incubated overnight at 4 °C with anti-FLAG antibody or, as a negative control, anti-Cucumber mosaic virus polyclonal antibody (DSMZ, Braunschweig, Germany), both at 1:1000 dilution. After washing, sections were incubated for 1 h at room temperature, with 15 nm gold-labelled goat anti-rabbit serum (1:20; British BioCell, Cardiff, UK) and stained with 2% uranyl acetate and lead citrate, before being examined with a 100SX TEM (Jeol, Japan) operating at 80 KV.

### Fluorescence microscopy

Leaves from Arabidopsis plants grown for 2 weeks in soil were cut in half lengthwise and primary veins were removed. Staining was with 3,3’-dihexyloxacarbocyanine (DiOC6, Molecular Probes) at a concentration of 0.5 μg ml^−1^ in PBS for 10 min, followed by washing three times in PBS. Small sections of stained leaves were placed on a microscope slide and visualized with a 63x oil immersion objective mounted on an Axiovert 200 microscope (Carl Zeiss) equipped for epifluorescence. Simultaneous visualization of DiOC6 stain (488 nm excitation/520 nm emission) and bright-field (visible lamp) was performed using the sequential scanning facility of the microscope. Images were assembled with Adobe Photoshop software 10.0.

## Results

### A proportion of 16γz present in maize PB is solubilized by alcohol

**Fig. 2.**
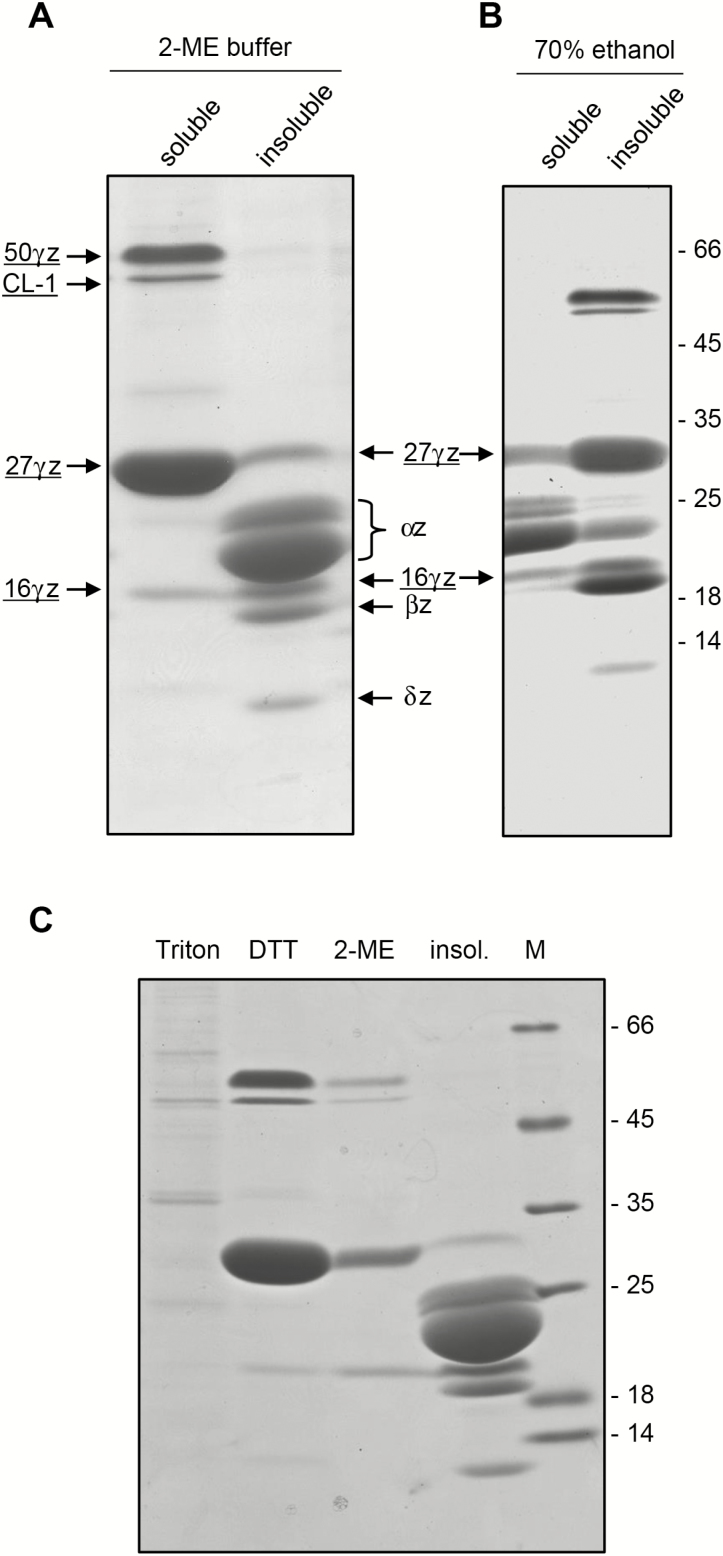
The solubility of 16γz accumulated in maize protein bodies (PBs) is intermediate between those of α-zeins and the other γ-zeins. PBs purified from maize seeds, collected at 25d after pollination, were treated at 4 °C with buffer containing 4% 2-ME (A) or at 25 °C with 70% ethanol in H_2_O (B). After centrifugation, soluble and insoluble proteins were analysed using SDS-PAGE and Coomassie staining. The different zein polypeptides (αz, βz, γz, δz) and CL-1 are indicated. Those whose identities were confirmed by LC-ESI-MS/MS are underlined (see Supplementary Fig. S1 and Supplementary Table S1). (C) Purified PBs were sequentially extracted with buffer containing 1% Triton X-100, 2 mM DTT, and 4% 2-ME. After each step, the suspension was centrifuged and the soluble material was analysed using SDS-PAGE and Coomassie staining, together with the insoluble material of the last extraction (insol.). The positions of molecular mass markers (M, in kD) are indicated to the right in (B) and (C).

16γz can be efficiently solubilized from maize endosperm by 70% ethanol supplied with reducing agent (Kim *et al.*, 2006), but its solubility in each of these agents alone is less clear. Treatment of purified PBs with buffer containing 2 mM DTT efficiently solubilizes 27γz and 50γz, but not PB polypeptides with molecular mass in the 16-kD range (Vitale *et al.*, 1982). We therefore examined in more detail the solubility of 16γz accumulated in maize. An endosperm PB fraction prepared using a sucrose gradient was first treated with buffer containing 4% 2-mercaptoethanol (2-ME buffer, Fig. 2A). This reducing buffer solubilizes recombinant 27γz (Mainieri *et al.*, 2014). The polypeptides that are underlined in Fig. 2 were identified by LC-ESI-MS/MS analysis (Supplementary Fig. S1 and its associated Methods, and Supplementary Table S1; 27γz and 16γz identities were confirmed in both the soluble and insoluble fractions); polypeptides without underlining indicate zeins identified solely based on their typical SDS-PAGE migration rates (notice that prolamins migrate more slowly than expected from their sequences). The 2-ME buffer very efficiently solubilized 27γz and 50γz (Fig. 2A), as expected (Vitale *et al.*, 1982). In contrast, solubilization of 16γz was only partial, with most of the protein remaining in the insoluble precipitate, unlike the two other γ-zeins (Fig. 2A). α-zeins, which are alcohol-soluble (Misra *et al.*, 1976), were efficiently solubilized by 70% ethanol at 25 °C (Fig. 2B). In addition, a significant proportion of 16γz was solubilized by ethanol, whereas 50γz and 27γz remained totally or almost totally insoluble (Fig. 2B). When PBs were sequentially extracted with buffer containing non-ionic detergent, 2 mM DTT (as in Vitale *et al.*, 1982) or 4% 2-ME, the results confirmed that γ-zeins are insoluble unless reduced and indicated that DTT was not more efficient than 2-ME in solubilizing 16γz (Fig. 2C).

Minor amounts of corn legumin-1 (CL-1), an 11S storage globulin (Woo *et al.*, 2001; Yamagata *et al.*, 2003), were extracted using non-reducing buffer containing non-ionic detergent, but most of this protein was extracted in the presence of a reducing agent (Fig. 2A–C, Supplementary Fig. S1). 11S storage proteins usually accumulate in protein storage vacuoles, but the presence of CL-1 in PBs, especially at late stages of endosperm maturation, has been observed previously (Arcalis *et al.*, 2010; Reyes *et al.*, 2011).

The solubility of 16γz accumulated in maize was therefore intermediate between that of α-zeins and the other γ-zeins, and distinct from that of CL-1, and β- and δ-zeins (these two minor zeins were not efficiently solubilized by either solvent, Fig. 2), indicating that 16γz may have specific polymerization properties. We verified this by comparing the destinies of 16γz and 27γz expressed individually in plant cells.

### Recombinant 16γz and 27γz are retained intracellularly but have different solubility

**Fig. 3.**
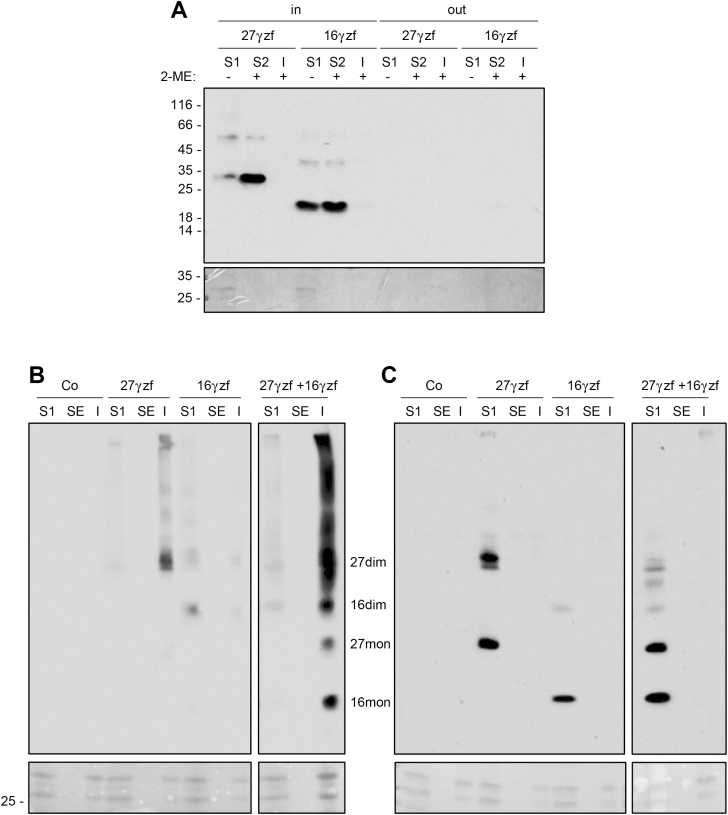
Recombinant 16γz and 27γz are retained intracellularly but have different solubility. Protoplasts were isolated from tobacco leaves and transiently transformed either with plasmids encoding the indicated constructs or with the empty vector (Co) and analysed after incubation for 20 h. (A) Protoplasts (in) or incubation medium (out) were homogenized in the absence (–) of 2-ME. After centrifugation, soluble (S1) and insoluble fractions were collected. The insoluble material was resuspended in the presence (+) of 2-ME and subjected to a second centrifugation, to obtain the new soluble (S2) and insoluble (I) fractions. (B) Protoplasts were homogenized in the absence of 2-ME. After centrifugation, soluble (S1) and insoluble fractions were collected. The insoluble material was resuspended with 70% ethanol and subjected to a second centrifugation, to obtain the new soluble (SE) and insoluble (I) fractions. (C) As in (B), but the first homogenization was performed in the presence of 4% 2-ME. In (A–C), the upper images show analysis of each fraction by SDS-PAGE and protein blotting with anti-FLAG antibody, whilst the lower images show Ponceau S staining. The positions of molecular mass markers are shown to the left, in kD. In (B, C) the positions of dimers (dim) and monomers (mon) of 27γzf (27) and 16γzf (16) are indicated.

The 16γz sequence was tagged at the C-terminus with the FLAG epitope. This construct (16γzf) and similarly tagged 27γz (27γzf; Mainieri *et al.*, 2014) were first transiently expressed in tobacco protoplasts. SDS-PAGE and protein blotting with anti-FLAG antibody performed ~20 h after transfection indicated that 16γzf was recovered intracellularly, with almost no sign of secretion (Fig. 3A). In addition to the expected abundant monomers, a small proportion of 16γzf was detected as what appear to be dimers and larger oligomers, not disassembled by the denaturation buffer. Both the lack of secretion and the incomplete disassembly by the SDS-PAGE denaturing/reducing buffer were also characteristic of 27γzf expressed in protoplasts (Fig. 3A, and see Mainieri *et al.*, 2014) and leaves of transgenic Arabidopsis (Geli *et al.*, 1994). Sequential extraction with non-reducing buffer and then buffer supplemented with 4% 2-ME indicated that 27γzf was almost completely insoluble unless reduced (Fig. 3A, S2 fraction), as previously established (Mainieri *et al.*, 2014). A significant proportion of 16γzf molecules was instead also soluble in the absence of reducing agent (Fig. 3A, S1 fraction), indicating inefficient formation of insoluble polymers. 70% ethanol did not solubilize either of the two constructs (Fig. 3B, SE fraction). When 27γzf and 16γzf were transiently co-expressed, both were almost completely insoluble in non-reducing buffer or 70% ethanol (Fig. 3B, I fraction). Therefore, the two γ-zeins interacted, and 27γzf had a dominant effect in inhibiting 16γzf solubility in the absence of reducing agent. When the first buffer of the sequential extraction was supplemented with 4% 2-ME, both individually expressed and co-expressed γ-zeins were fully solubilized, confirming the role of disulfide bonds in determining insolubility (Fig. 3C, S1 fraction). These data were consistent with the insolubility of 16γz when natural maize PBs were treated with non-reducing buffer (see Fig. 2C) and suggested that its partial solubility in ethanol was due to interactions with α-zeins. The relative proportions of monomers and oligomers detected by SDS-PAGE varied in independent experiments, but their different solubility in non-reducing conditions, when individually expressed, was consistently observed (compare Fig. 3A and B, and see also Supplementary Fig. S2).

### In transgenic Arabidopsis, 16γzf is mostly unable to assemble into subcellular structures with the typical PB density

**Fig. 4.**
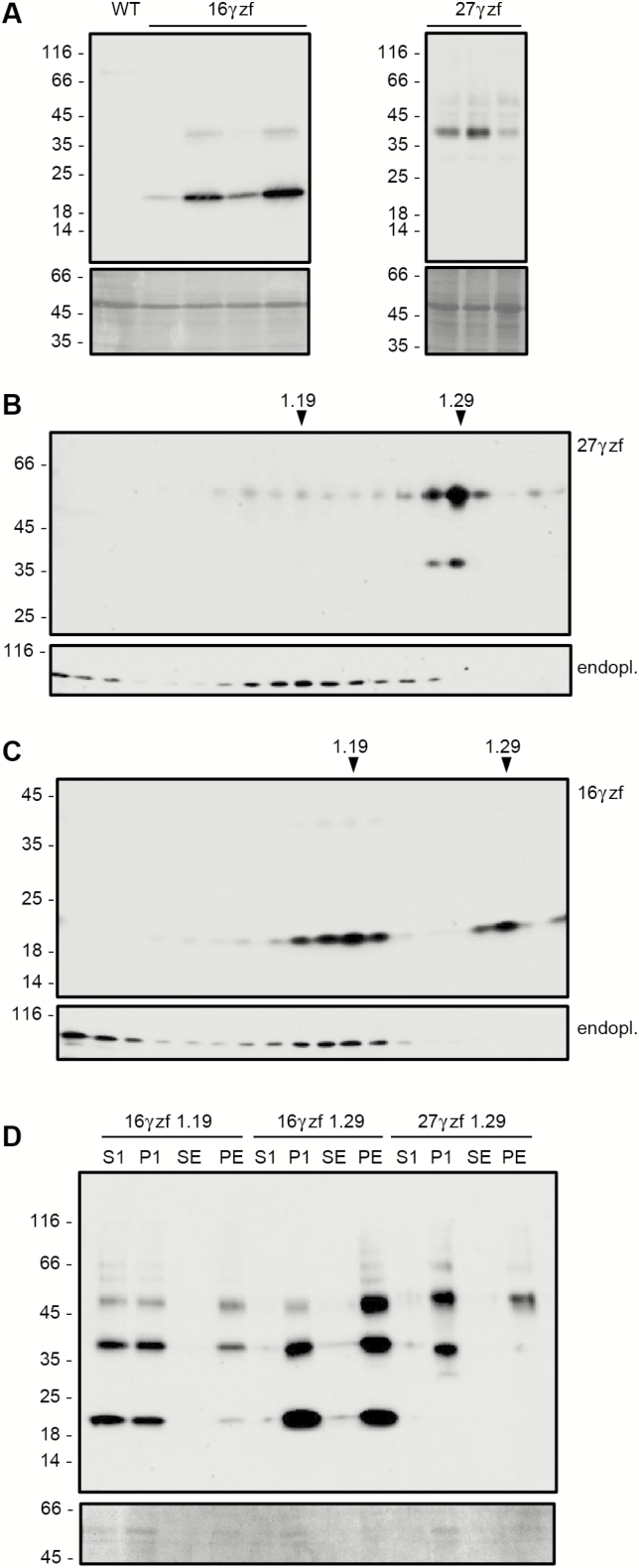
Assembly of 16γzf into dense subcellular structures is inefficient. (A) Leaves from transgenic Arabidopsis expressing 27γzf or 16γzf, or from wild-type plants (WT) were homogenized in the presence of 2-ME. Soluble proteins were analysed using SDS-PAGE. Each individual lane represents an independent transgenic plant. The upper images are protein blots with anti-FLAG antibody; lower images are Ponceau S staining. (B, C) Leaves from transgenic Arabidopsis expressing 27γzf (B) or 16γzf (C) were homogenized in the presence of 12% (w/w) sucrose and in the absence of detergent. The homogenates were fractionated by ultracentrifugation on 16–65% (w/w) isopycnic sucrose gradients. Proteins in each gradient fraction were analysed by SDS-PAGE and protein blotting, with anti-FLAG (27γzf, 16γzf) antibody or anti-endoplasmin (endopl.) serum. The top of gradients are at the left and the numbers at the top indicate the density (g ml^−1^). (D) Fractions around either 1.19 or 1.29 density from the gradients shown in (B, C) were pooled, extracted with buffer without reducing agent and centrifuged. Supernatants (S1) and pellets (P1) were collected. An aliquot of P1 was further treated with 70% ethanol and centrifuged to obtain ethanol-soluble (SE) and insoluble (PE) material. The upper image shows analysis by SDS-PAGE and protein blotting with anti-FLAG antibody; the lower image shows Ponceau S staining. In (A–D) the numbers at the left indicate the positions of molecular mass markers (kD).

To compare the long-term destinies of the two zeins, the tagged constructs were expressed in transgenic Arabidopsis under a constitutive promoter. These plants did not show visually evident phenotypes or clear alterations in growth and reproduction. For each construct, accumulation in leaves varied in different independent transgenic plants, but the electrophoretic pattern was unaffected by the level of final accumulation (Fig. 4A). 16γzf showed the same electrophoretic patterns whether extracted from transgenic leaves or transiently transfected protoplasts, while most 27γzf monomers were clearly of higher apparent molecular mass in transgenic leaves (around 40 kD), with only a minor proportion migrating as in transient expression (around 30 kD; compare Figs 3A and 4A). This indicated 27γzf-specific post-translational modifications that were not yet detectable during the first hours after synthesis, and that did not occur in maize seeds. Hydroxylation of proline residues is the most likely explanation, as previously observed (Geli *et al.*, 1994; Mainieri *et al.*, 2014).

Subcellular localization was first investigated by isopycnic ultracentrifugation of homogenates prepared in the absence of detergent, to maintain membrane integrity. 27γzf accumulated mainly in structures with density around 1.29 (Fig. 4B). This is consistent with the known ability of 27γz to form homotypic PBs in the absence of the other zeins (Geli *et al.*, 1994; Coleman *et al.*, 1996) and the known high density of zein or zeolin PBs in maize or transgenic plants (Larkins and Hurkman, 1978; Geli *et al.*, 1994; Mainieri *et al.*, 2004). Much lower amounts of 27γzf, probably constituted by newly synthesized molecules not yet assembled into dense PBs, were recovered in lighter subcellular fractions that contain the ER resident endoplasmin (Klein *et al.*, 2006) and have the typical ER density (Fig. 4B). 16γzf was similarly present in the two distinct subcellular fractions, but most of the protein was in this case in the endoplasmin-containing ER, suggesting a poor ability to form PBs (Fig. 4C).

To determine the solubility of 16γzf or 27γzf present at the two positions along the gradient, fractions around 1.19 or 1.29 density were pooled, extracted with buffer without reducing agent, and centrifuged to separate soluble and insoluble proteins. Around 50% of 16γzf present in the less-dense fraction was solubilized by this treatment (Fig. 4D, S1), whereas nearly 100% of 16γzf or 27γzf present in fractions at 1.29 density was insoluble (Fig. 4D, P1). Treatment of P1 with 70% ethanol did not solubilize 16γzf or 27γzf (Fig. 4D, SE and PE; note that treatment with ethanol makes the denaturation of oligomers more difficult). We concluded that the relevant proportion of 16γzf that was not assembled into dense subcellular structures was in part also soluble in the absence of reducing agent, but no 16γzf molecules insoluble in aqueous buffer were alcohol-soluble. This strongly suggested that 16γz in maize PBs is partially alcohol-soluble due to association with alcohol-soluble α-zeins, as also suggested by the data in Fig. 3B.

**Fig. 5.**
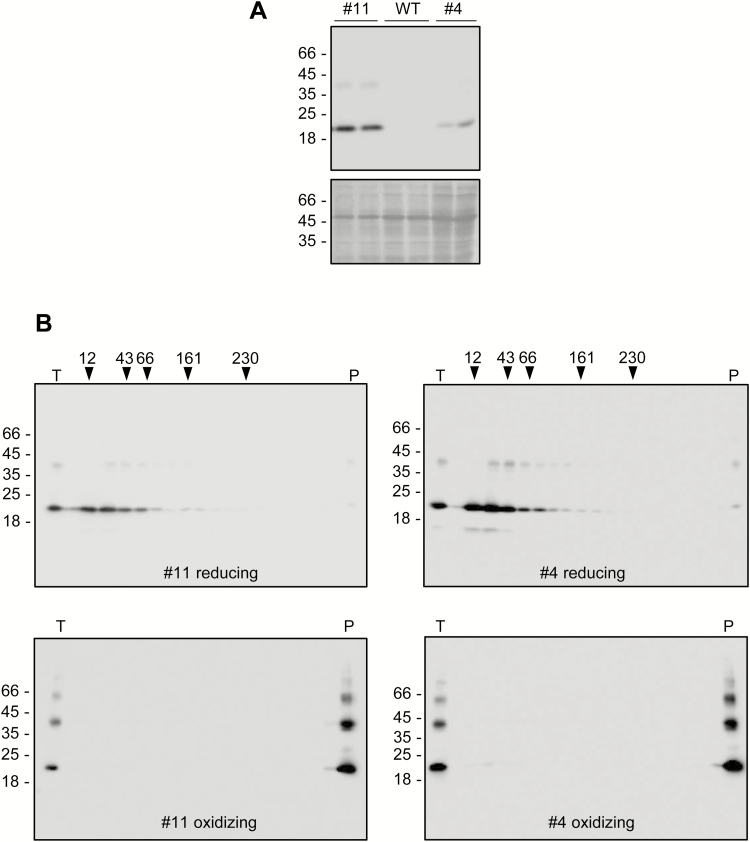
16γzf forms large, disulfide-dependent polymers. (A) Homogenates were prepared from leaves of two independent transgenic Arabidopsis lines that accumulate different amounts of 16γzf (#11 and #4, two plants for each line), and from leaves of untransformed wild-type Arabidopsis (WT), and analysed using SDS-PAGE. The upper image shows the protein blot with anti-FLAG antibody; the lower image shows Ponceau S staining. (B) Homogenates were prepared in either oxidizing or reducing buffer, and fractionated by velocity gradient ultracentrifugation. The top of each gradient is at the left. T, unfractionated total homogenate; P, pellet at the bottom of the tube after centrifugation. The numbers at the top indicate the positions where molecular mass markers migrate along the gradients. In (A, B) the numbers at the left indicate the positions of SDS-PAGE molecular mass markers (kD).

When homogenates, prepared in non-reducing buffer supplemented with non-ionic detergent, were subjected to velocity sucrose-gradient ultracentrifugation, both 27γzf and zeolin migrated at the bottom of tubes, indicating that they are large polymers (Mainieri *et al.*, 2004, 2014). Given the partial different subcellular localization and solubility of 16γzf with respect of 27γzf, we investigated whether 16γzf also forms large polymers held together by disulfide bonds. Two plants accumulating different amounts of 16γzf were analysed, to verify whether the expression levels influenced oligomerization (Fig. 5A). 16γzf migrated to the bottom of the velocity ultracentrifugation tubes, independently of its level of accumulation (Fig. 5B, bottom panels). When leaf homogenization and velocity centrifugation were performed in reducing conditions, 16γzf migrated in a position corresponding to monomers (Fig. 5B, top panels). We concluded that 16γzf forms extensive, disulfide-dependent polymers, in spite of its poor ability to form high-density subcellular compartments. We therefore used electron microscopy to compare the subcellular structures formed by 27γzf and 16γzf.

### 16γzf polymerizes into unusual reticular threads that markedly alter ER morphology

**Fig. 6.**
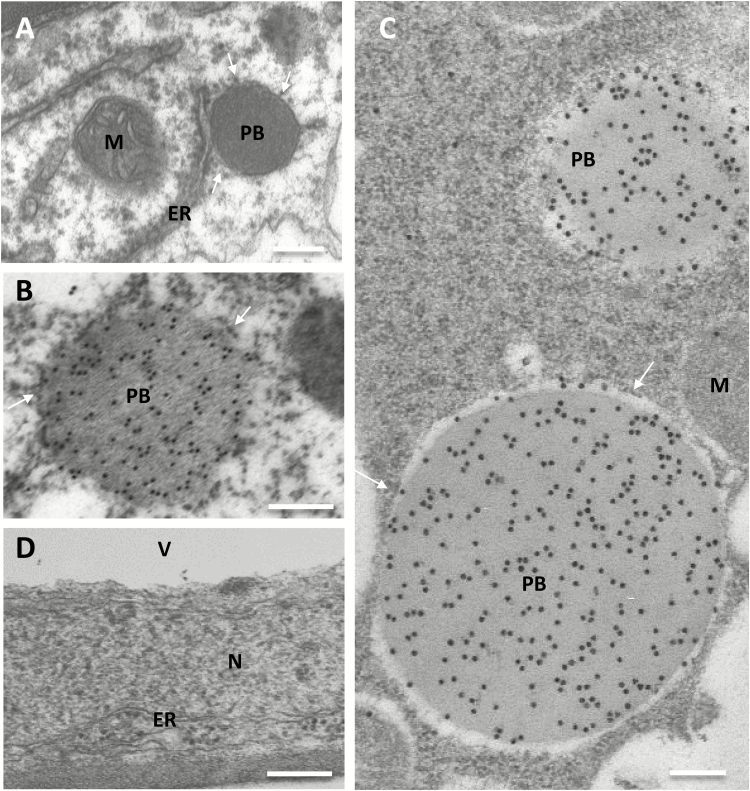
27γzf forms protein bodies (PBs). Leaves from 6-week-old transgenic Arabidopsis plants expressing 27γzf (A–C) or wild-type plants (D) were examined using electron microscopy. (A, D) Ultrathin sections post-fixed with osmium. (B, C) Immunolabelling with anti-FLAG antibody and secondary 15-nm gold-conjugated goat anti-rabbit serum. ER, endoplasmic reticulum; PB, protein body; M, mitochondria; N, nucleus, V, vacuole. Arrows indicate ribosomes. Note that in non-osmicated immunolabelled tissues (B, C) ER membranes are not detectable; however, numerous ribosomes are visible aligned outside the PB periphery (arrows). Scale bars are 200 nm.

In addition to typical ER membranes (Fig. 6A, ER, compare with wild-type tissue in 6D), 27γzf leaf tissue showed electron-dense, round structures with diameters from a few hundred nanometres to more than one micron, with attached ribosomes (Fig. 6, PB). These structures, not present in wild-type plants, were labelled by anti-FLAG antibody (Fig. 6B, C), thus indicating that 27γzf formed PBs. Homotypic PBs formed by recombinant 27γz have been observed in Arabidopsis vegetative tissues (Geli *et al.*, 1994) and tobacco seeds (Coleman *et al.*, 1996), although with smaller sizes than those that we observed.

**Fig. 7.**
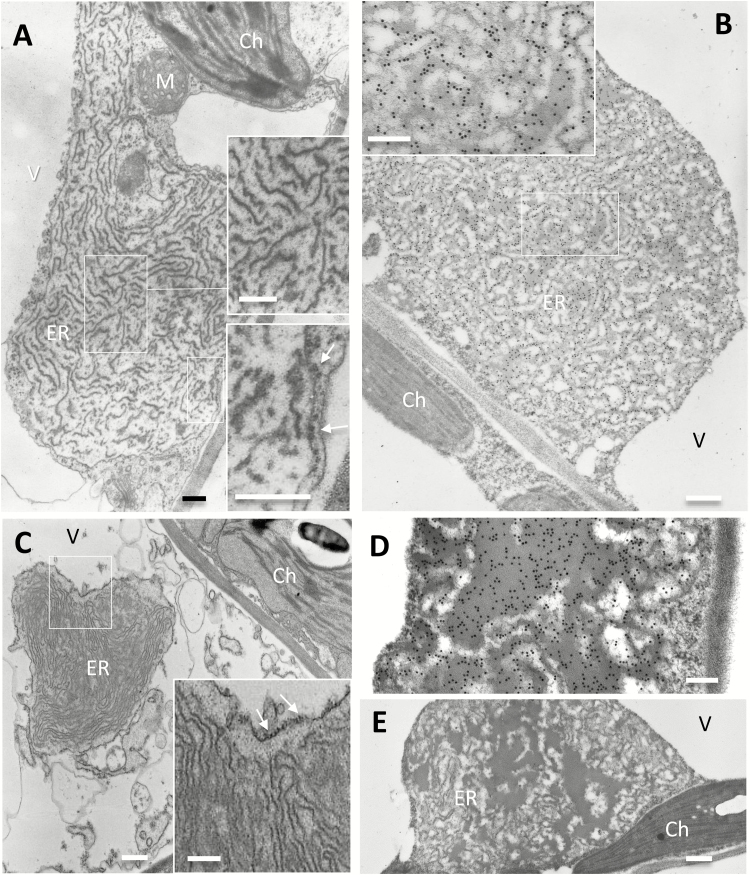
16γzf does not form protein bodies (PBs) but forms electron-dense structures in highly enlarged endoplasmic reticulum (ER) lumen. Leaves from 6-week-old transgenic Arabidopsis plants accumulating 16γzf in high (A, B, D, E) or low (C) amounts were analysed. (A, C) Ultrathin sections post-fixed with osmium. (B, D, E) Immunolabelling with anti-FLAG antibody (B, D) or irrelevant antibody as a negative control (E), and secondary 15-nm gold-conjugated goat anti-rabbit serum. The insets in (A–C) show magnifications to better illustrate the ribosomes attached on the cytosolic side of the ER membrane (arrows) and the electron-dense convoluted structures within the ER lumen. Ch, chloroplasts; M, mitochondria; V, vacuole. Scale bars in (A, D) and all insets are 200 nm; bars in (B, C, E) are 500 nm.

Markedly different structures were formed by 16γzf (Fig. 7). Large, irregular dilatations enclosed by a single membrane, often several micrometres wide, were detected (Fig. 7A; in Supplementary Fig. S3 black arrowheads mark the margins of this dilatation). The boundary membrane was surrounded by ribosomes (arrows in Fig. 7A, lower enlarged inset, and 7C, enlarged inset) and connections with tubular ER were occasionally seen (white arrowheads in Supplementary Fig. S3). The vacuole was often pressing against these dilatations, sometimes leaving space for a thin layer of cytoplasm outside the dilated ER (visible in the post-fixed sample in Fig. 7A, and more easily seen in Supplementary Fig. S3 where the ER membrane is indicated). The lumen of the ER dilatations contained very extensive electron-dense structures of two forms: very electron-opaque, osmiophilic threads of various lengths and irregular orientation (well represented in Fig. 7A, C) were mainly observed, whereas a minor proportion formed more compact irregular structures of lighter electron-density (Fig. 7B, enlarged inset, and more evident in Fig. 7D, E). In non-osmicated tissues immunolabelled with anti-FLAG antibody, the convolutions appeared less sharp; however, gold particles were mostly aligned on them (Fig. 7B, D). No labelling occurred using an irrelevant antibody, confirming that the structures were formed by 16γzf (Fig. 7E). The relative abundance of the two types of structures was variable in different ER dilatations, but when independent transgenic plants accumulating high (Fig. 7A, B, D, E) or low (Fig. 7C) amounts of 16γzf were compared, no clear relationship between recombinant protein abundance and the type of 16γzf structure could be established.

**Fig. 8.**
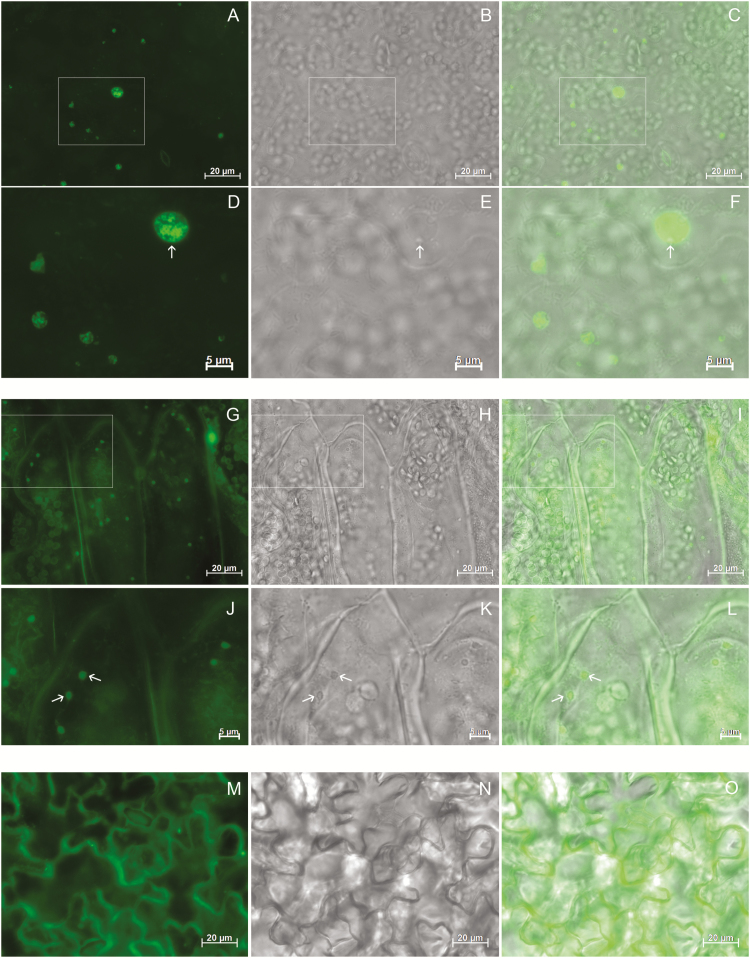
27γzf protein bodies (PBs) and 16γzf endoplasmic reticulum (ER) enlargements can be detected by fluorescence staining of the ER. Leaf tissue from 16γzf (A–F), 27γzf (G–L) or wild-type (M–O) Arabidopsis plants was stained with DiOC6 dye and examined using epifluorescence microscopy. (A, D, G, J, M): DiOC6 fluorescence (green); (B, E, H, K, N): bright-field; (C, F, I, L, O): merged images. Camera exposure time (ms): 61 (A), 502 (G), 8352 (M). Boxes in (A–C) and (G–I) indicate the regions that are shown at higher magnification in (D–F) and (J–L), respectively. Arrows indicate enlarged ER (D–F) or PBs (J–L).

The ER vital lipophilic dye DiOC6 also efficiently stains PBs in developing endosperm cells in both rice and maize, probably due to its high affinity for the hydrophobic prolamin polypeptides (Muench *et al.*, 2000; Washida *et al.*, 2004). To complement the observations of electron microscopy, leaves were incubated with DiOC6 and observed under conventional fluorescence microscopy (Fig. 8). In 16γzf leaves, DiOC6 highlighted enlarged structures of various sizes. Higher magnification (Fig. 8A, inset, and magnification in 8D) showed that their content was not uniform, consistent with the structures observed by electron microscopy (Fig. 8D, arrow and compare with Fig. 7). In 27γzf leaves, more uniformly stained PBs with the classical size and round morphology were visible, as expected (Fig. 8G, and arrows in 8J). Structures similar to those in 16γzf and 27γzf were not detected in wild-type tissue, even at very high camera exposure times that highlighted the cell periphery, as expected for the ER lipophilic dye (Fig. 8M). Both the 16γzf structures and the 27γzf PBs were also detected under transmitted light (Fig. 8E, K, arrows).

We concluded that 16γzf, unlike 27γz, is unable to form PBs and instead polymerizes into novel electron-dense structures that mostly appear as irregular threads and cause marked enlargement of the ER lumen.

### The N-terminal domain of 16γzf is responsible for the inefficient formation of insoluble polymers

**Fig. 9.**
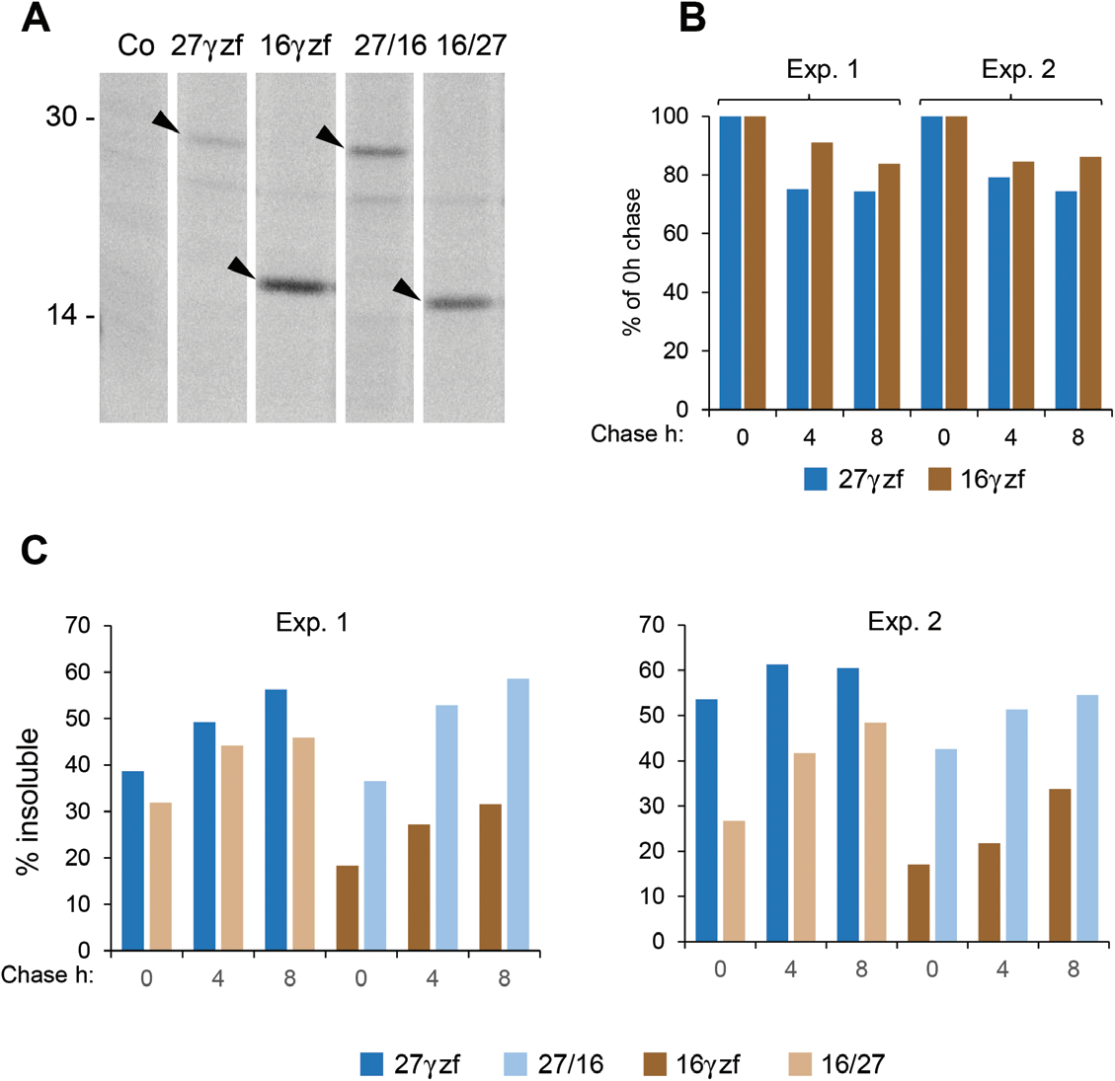
The N-terminal domain of 16γzf has a major role in inhibiting the formation of insoluble polymers. Protoplasts prepared from tobacco leaves were transiently transfected either with plasmids encoding the indicated constructs or with empty plasmids (Co). (A) Transfected protoplasts were pulse-labelled with radioactive amino acids for 1 h before homogenization in reducing conditions. Proteins were immunoselected using anti-FLAG antibody and analysed using SDS-PAGE and radiography. The different lanes are from a single exposure of a single radiograph from which irrelevant lanes have been removed. The newly synthesized recombinant polypeptides (arrowheads) and the positions of molecular mass markers (numbers at left, kD) are indicated. (B) Protoplasts pulse-labelled as in (A) were subjected to chase for the indicated time (h), homogenized in the presence of 2-ME, immunoselected with anti-FLAG antibody, and analysed using SDS-PAGE and radiography. At each chase time-point, the densities of the relevant radioactive bands were quantified and expressed as percentage of the intensity at 0 h chase. (C) Protoplasts pulse-labelled as in (A) and chased for the indicated time (h) were then subjected to sequential homogenization steps, first in the absence 2-ME and then treating the insoluble material with 2-ME. Proteins at each step were immunoselected with anti-FLAG antibody and analysed using SDS-PAGE and radiography. At each time-point, the densities of the relevant radioactive bands were quantified and, for each construct, expressed as percentage in the second immunoprecipitation step divided by the sum of the two immunoprecipitations (% insoluble). In (B, C) values from two fully independent transient expression experiments are shown.

To identify the structural features of 16γz that did not allow efficient formation of insoluble polymers, we measured the loss of solubility during pulse-chase labelling in transiently transfected tobacco protoplasts. After pulse labelling for 1 h with a mixture of [^35^S]Met and [^35^S]Cys, protoplasts were subjected to chase for 0, 4, or 8 h. At each time-point, protoplasts were directly extracted in reducing conditions (thus solubilizing all molecules of each construct, to measure synthesis and stability; Fig. 9A, B) or sequentially extracted: first in non-reducing buffer and then treating the insoluble material with reducing buffer (to calculate at each time-point the percentage of molecules that are insoluble unless reduced, Fig. 9C). Each extract was immunoselected with anti-FLAG antibody and analysed using SDS-PAGE and radiography. Newly synthesized 16γzf and 27γzf had the expected molecular mass (Fig. 9A). 16γzf was slightly more stable during the chase (Fig. 9B; data are from two fully independent experiments). Already at the 0 h chase time-point, a much higher percentage of 27γzf than 16γzf was insoluble unless reduced (Fig. 9C). Insolubility increased during the chase, but the marked difference between the two zeins remained, as expected from the previous solubility assays (Figs 3, 4D). To map the insolubility determinant, we prepared two constructs, 27/16 and 16/27, in which the N-terminal domain of each zein was exchanged with that of the other (Fig. 1, the green arrowheads indicate the points of exchange). Since most of the molecular mass difference between the two zeins is due to their N-terminal domain, the SDS-PAGE migrations of 27/16 and 16/27 are similar to those of 27γzf and 16γzf, respectively (Fig. 9A). The replacement of the natural N-terminal domain of 27γzf with that of 16γzf markedly inhibited insolubilization (Fig. 9C, compare 27γzf and 16/27), whereas the reciprocal replacement markedly stimulated this process (Fig. 9C, compare 16γzf and 27/16). This indicated that the N-terminal domain is the major determinant for the different behavior of the two zeins.

## Discussion

Mutations and insertions in the ancient seed storage proteins of the 2S albumin class were the first events in the origination of prolamins (Xu and Messing, 2009; Gu *et al.*, 2010; Pedrazzini *et al.*, 2016). This led to the assembly in PBs and a change in the subcellular compartment used for permanent accumulation from the vacuole to the ER, particularly in rice and panicoid cereals such as maize, sorghum, and millet (Lending and Larkins, 1989; Shewry and Halford, 2002; Saito *et al.*, 2012).

16γz originated upon maize WGD (Xu and Messing, 2008) and it is mainly characterized by deletions in the N-terminal region of 27γz, the most ancient γ-zein. We have shown here that recombinant 16γzf ectopically expressed in vegetative tissues accumulated within the ER, forming unusual structures. These did not resemble PBs or other ER-located polymers formed by natural or recombinant proteins expressed in plants (Bagga *et al.*, 1995; Mainieri *et al.*, 2004; de Virgilio *et al.*, 2008; Conley *et al.*, 2009; Saito *et al.*, 2009; Torrent *et al.*, 2009; Llop-Tous *et al*, 2010). 16γzf structures mainly consisted of extensive, convoluted but well-defined filamentous threads; more rarely, the enlarged ER also contained irregular, homogenously electron-dense sectors, which may represent the proportion of 16γzf that had become insoluble. At the onset of prolamin accumulation, initial irregular dilatations along the ER have been observed in rice, but with diameters below 1 μm (Kawagoe *et al.*, 2005). 8S globulin, a mung bean vacuolar storage protein, forms 0.2–0.6 μm ER enlargements in transgenic tobacco BY2 cells and, as a GFP fusion, in Arabidopsis vegetative tissues and young developing seeds, to be correctly deposited in Arabidopsis storage vacuoles only at later seed development (Wang *et al.*, 2013). The sizes of these ER structures are one order of magnitude smaller compared the dilatations caused by 16γzf. Wider, irregular ER enlargements are formed by the expression of the N-terminal region of 27γz in Arabidopsis, but these have homogeneous electron density, with no signs of filaments (Geli *et al.*, 1994). PBs formed by chimeric fusions containing spider elastin-like polypeptides can have a loosely packed content, but they are round and rarely larger than 3 μm, with no well-defined filaments (Conley *et al.*, 2009; Phan *et al.*, 2014). Thus, the unusual structures that we found formed by 16γzf markedly differed from 27γzf PBs and from ER enlargements formed by various other storage proteins at early stages of seed development or by protein fusions that polymerize in the ER.

However, 16γzf threads strikingly resemble those formed by diabetes insipidus-inducing mutants of the antidiuretic hormone arginine vasopressin precursor (Birk *et al.*, 2009; Beuret *et al.*, 2017). These dominant mutations can be in different locations along the precursor, but they all result in abnormal inter-chain disulfide bonds leading to oligomerization and, in some cases, partial resistance to denaturation by SDS/reducing agent, whereas the normal precursor has eight intra-chain bonds. The misfolded precursors thus accumulate in the ER instead of trafficking to secretory granules and form irregularly packed electron-dense filaments, which in some cases coalesce in more uniformly electron-dense regions, similar to 16γzf. Although the mutated precursors seem unable to form canonical amyloid cross-β-sheets, their ability to form fibers resembles amyloid aggregation (Beuret *et al.*, 2017).

Our results indicated that 16γzf was not a structurally defective protein rapidly degraded by ER quality control. 16γzf threads were disulfide-bonded polymers that remained partially soluble in oxidizing conditions, unlike 27γzf polymers. Only when the two recombinant zeins were co-expressed, 16γzf become fully insoluble unless reduced, indicating direct interactions with 27γzf. 16γz present in natural maize PBs was in part solubilized by alcohol together with α-zeins, but no alcohol-soluble 16γzf was detected in transgenic Arabidopsis or upon transient co-expression with 27γzf. This supports the hypothesis that, in maize, at least one of the alcohol-soluble α-zeins directly interacts with16γz, consistent with the location of 16γz in natural PBs (Lending and Larkins, 1989; Yao *et al.*, 2016) and the results of yeast two-hybrid assays (Kim *et al.*, 2002, 2006). A specific role of 16γz in natural, heterotypic PB assembly is also supported by the characteristics of two maize mutations with opaque endosperm, *mucronate* and *opaque10*. *mucronate* is a frameshift mutation that completely changes the 16γz sequence for the last 63 amino acids, abolishes its solubility in 70% ethanol supplemented with 2-ME, and markedly weakens the interaction with 22-kD α-zein (Kim *et al.*, 2006). In *mucronate* seeds, the overall amount of zeins is reduced (Salamini *et al.*, 1983) and PBs have angular deformations that often interrupt the outer layer, indicating defects in the organization of the interface between α- and γ-zeins (Zhang and Boston, 1992). *opaque10* is a frameshift mutation generating a premature stop codon in a cereal-specific protein located in PBs (Yao *et al.*, 2016). *opaque10* PBs are misshaped and often irregularly elongated. The ordered localizations of 16γz and of the 22-kD α-zein that is normally located next to it are disrupted, and the two zeins are dispersed in the PB (Yao *et al.*, 2016). RNAi, used to inhibit the synthesis of γ-zeins in maize, also causes PB misshaping and angular deformations (Wu and Messing, 2010). A specific role of 16γz could not be established in this case, since the synthesis of both the 27- and 16-kD polypeptides was almost fully inhibited. However, RNAi in which the synthesis of 16γz, 50γz, and β-zein was concomitantly suppressed indicated that these proteins are mainly involved in PB expansion, whereas 27γ-zein controls PB initiation and shape, consistent with our data in transgenic Arabidopsis (Guo *et al.*, 2013).

Sorghum (*Sorghum bicolor*), a very close relative of maize (Swigoňová *et al.*, 2004), has not undergone WGD and contains only two genes belonging to the same prolamin II group of γ-zeins, *kafirin2γ27* and *kafirin2γ50* (Belton *et al.*, 2006; Xu and Messing, 2009), and is therefore lacking a 16γz orthologue. Similar to β- and γ-zeins, β- and γ-kafirins form the more electron-dense structures of the PB; however, these are not limited to the PB periphery and are also concentrated in the central core or form patches within the less-dense regions (Shull *et al.*, 1992). This lack of organization in layers with clear boundaries between dense and less-dense regions (the latter mainly containing α-type prolamins), compared to maize PBs may thus be related to the absence of a 16γz-like prolamin.

Our domain-exchange results suggested that the different behavior of the two γ-zeins was mainly due to their N-terminal domains. A synthetic version of the (VHLPPP)_8_ repeated segment has an amphipathic polyproline II structure and *in vitro* affinity to liposomes that partially mimics the lipid composition of the plant ER, suggesting that the repeat may favor interaction of 27γz with the inner surface of the ER membrane (Kogan *et al.*, 2004). The Zera sequence is a 27γz portion almost identical to the one used to construct zeolin and, like zeolin, it determines PB formation in a Cys-dependent fashion when fused to a number of proteins (Torrent *et al.*, 2009). In a Zera-fluorescent protein fusion, progressive deletion of the Pro-rich hexapeptides leads to progressively increased secretion and reduced PB size but does not alter their spherical shape (Llop-Tous *et al.*, 2010), indicating that the peculiar structures formed by 16γz are not simply due to the loss of repeats. Indeed, the N-terminal region of 16γz has also lost three Cys residues and contains two degenerated Pro-rich sequences containing two new Tyr residues—aromatic amino acids inhibit the formation of polyproline II helices (Brown and Zondlo, 2012)—as well as other aromatic amino acids and a new Gln-rich short sequence (Fig. 1). In combination, these features may have abolished the ability to interact orderly with lipids and determined the formation of rod-like polymers involved in stabilizing the γ-zein/α-zein interface.

Proteins containing disulfide bonds generally have higher evolutionary rates (Feyertag and Alvarez-Ponce, 2017). Intra-chain disulfides probably stabilize important conformations and thus have a buffering, chaperone-like effect that makes the polypeptide more tolerant to mutations; thus, once acquired, inter-chain disulfides are rarely changed (Wong *et al.*, 2011; Feyertag and Alvarez-Ponce, 2017). Unpaired Cys residues are also relatively more conserved than other amino acids (Wong *et al.*, 2011). The major deletion and the mutations generating 16γz have eliminated a number of 27γz cysteine residues and have altered the biochemical and polymerization properties of the prolamin, but they have not caused gross misfolding and degradation by quality control. They have instead promoted a new role of the protein and a new PB organization.

Prolamins form peculiar heteropolymers. Analysis of many prolamin polypeptides and their positioning within a PB in different grasses indicates that a high genetic variability is tolerated, probably because PB function is simply constituted by the high accumulation of reduced nitrogen in the first compartment of the secretory pathway. However, within an individual species, certain requirements for optimal PB assembly exist, as indicated by the many natural and artificial cereal mutants analysed. We have shown here that an apparently defective zein polypeptide, generated upon maize whole-genome duplication, forms very unusual structures that may explain its specific structural role at the interface between the ancient and the more recently evolved maize prolamins. The organization of 16γz structures resembles abnormally disulfide-linked, amyloid-like fibers formed by pathological mutants of a human hormone precursor. It thus appears that mutations giving rise to similar abnormal structures within the ER can result in pathogenic loss of function in one case but can be exploited in a developmental process in another.

## Supplementary data

Supplementary data are available at *JXB* online.

Fig. S1. Identities of the major polypeptides present in purified maize PBs, as determined by LC-ESI-MS/MS analysis (including the protocol for protein in-gel digestion and the analysis).

Fig. S2. Variability in denaturation-resistant oligomers.

Fig. S3. Dilated ER in leaf cells of transgenic Arabidopsis expressing 16γzf.

Table S1. Peptide identification by LC-ESI-MS/MS analysis, and protein assignment.

Supplementary Materials

## References

[CIT0001] Arcalis E, Stadlmann J, Marcel S, Drakakaki G, Winter V, Rodriguez J, Fischer R, Altmann F, Stoger E 2010 The changing fate of a secretory glycoprotein in developing maize endosperm. Plant Physiology 153, 693–702. 20388665 10.1104/pp.109.152363 PMC2879800

[CIT0002] Bagga S, Adams H, Kemp JD, Sengupta-Gopalan C 1995 Accumulation of 15-kilodalton zein in novel protein bodies in transgenic tobacco. Plant Physiology 107, 13–23. 12228338 10.1104/pp.107.1.13 PMC161159

[CIT0003] Belton PS, Delgadillo I, Halford NG, Shewry PR 2006 Kafirin structure and functionality. Journal of Cereal Science 44, 272–286.

[CIT0004] Beuret N, Hasler F, Prescianotto-Baschong C, Birk J, Rutishauser J, Spiess M 2017 Amyloid-like aggregation of provasopressin in diabetes insipidus and secretory granule sorting. BMC Biology 15, 5. 28122547 10.1186/s12915-017-0347-9 PMC5267430

[CIT0005] Birk J, Friberg MA, Prescianotto-Baschong C, Spiess M, Rutishauser J 2009 Dominant pro-vasopressin mutants that cause diabetes insipidus form disulfide-linked fibrillar aggregates in the endoplasmic reticulum. Journal of Cell Science 122, 3994–4002. 19825939 10.1242/jcs.051136

[CIT0006] Brown AM, Zondlo NJ 2012 A propensity scale for type II polyproline helices (PPII): aromatic amino acids in proline-rich sequences strongly disfavor PPII due to proline–aromatic interactions. Biochemistry 51, 5041–5051. 22667692 10.1021/bi3002924

[CIT0007] Chapman BA, Bowers JE, Feltus FA, Paterson AH 2006 Buffering of crucial functions by paleologous duplicated genes may contribute cyclicality to angiosperm genome duplication. Proceedings of the National Academy of Sciences, USA 103, 2730–2735. 10.1073/pnas.0507782103 PMC1413778 16467140

[CIT0008] Clough SJ, Bent AF 1998 Floral dip: a simplified method for *Agrobacterium‐*mediated transformation of *Arabidopsis thaliana*. The Plant Journal 16, 735–743. 10069079 10.1046/j.1365-313x.1998.00343.x

[CIT0009] Coleman CE, Herman EM, Takasaki K, Larkins BA 1996 The maize gamma-zein sequesters alpha-zein and stabilizes its accumulation in protein bodies of transgenic tobacco endosperm. The Plant Cell 8, 2335–2345. 8989886 10.1105/tpc.8.12.2335 PMC161356

[CIT0010] Conley AJ, Joensuu JJ, Menassa R, Brandle JE 2009 Induction of protein body formation in plant leaves by elastin-like polypeptide fusions. BMC Biology 7, 48. 19664215 10.1186/1741-7007-7-48 PMC3224952

[CIT0011] de Virgilio M, De Marchis F, Bellucci M, Mainieri D, Rossi M, Benvenuto E, Arcioni S, Vitale A 2008 The human immunodeficiency virus antigen Nef forms protein bodies in leaves of transgenic tobacco when fused to zeolin. Journal of Experimental Botany 59, 2815–2829. 18540021 10.1093/jxb/ern143 PMC2486477

[CIT0012] Ems-McClung SC, Benmoussa M, Hainline BE 2002 Mutational analysis of the maize gamma zein C-terminal cysteine residues. Plant Science 162, 131–141.

[CIT0013] Faoro F, Tornaghi R, Belli G 1991 Localization of the Closteroviruses on grapevine thin sections and their identification by immunoglod labelling. Journal of Phytopathology 133, 297–306.

[CIT0014] Feyertag F, Alvarez-Ponce D 2017 Disulfide bonds enable accelerated protein evolution. Molecular Biology and Evolution 34, 1833–1837. 28431018 10.1093/molbev/msx135

[CIT0015] Geli MI, Torrent M, Ludevid D 1994 Two structural domains mediate two sequential events in [gamma]-zein targeting: protein endoplasmic reticulum retention and protein body formation. The Plant Cell 6, 1911–1922. 12244234 10.1105/tpc.6.12.1911 PMC160571

[CIT0016] Gomez-Navarro N, Miller E 2016 Protein sorting at the ER–Golgi interface. The Journal of Cell Biology 215, 769–778. 27903609 10.1083/jcb.201610031 PMC5166505

[CIT0017] Gu YQ, Wanjugi H, Coleman-Derr D, Kong X, Anderson OD 2010 Conserved globulin gene across eight grass genomes identify fundamental units of the loci encoding seed storage proteins. Functional & Integrative Genomics 10, 111–122. 19707805 10.1007/s10142-009-0135-x

[CIT0018] Guo X, Yuan L, Chen H, Sato SJ, Clemente TE, Holding DR 2013 Nonredundant function of zeins and their correct stoichiometric ratio drive protein body formation in maize endosperm. Plant Physiology 162, 1359–1369. 23677936 10.1104/pp.113.218941 PMC3707540

[CIT0019] Jiao Y, Wickett NJ, Ayyampalayam S, et al 2011 Ancestral polyploidy in seed plants and angiosperms. Nature 473, 97–100. 21478875 10.1038/nature09916

[CIT0020] Kassahn KS, Dang VT, Wilkins SJ, Perkins AC, Ragan MA 2009 Evolution of gene function and regulatory control after whole-genome duplication: comparative analyses in vertebrates. Genome Research 19, 1404–1418. 19439512 10.1101/gr.086827.108 PMC2720184

[CIT0021] Kawagoe Y, Suzuki K, Tasaki M, Yasuda H, Akagi K, Katoh E, Nishizawa NK, Ogawa M, Takaiwa F 2005 The critical role of disulfide bond formation in protein sorting in the endosperm of rice. The Plant Cell 17, 1141–1153. 15749763 10.1105/tpc.105.030668 PMC1087992

[CIT0022] Kim CS, Gibbon BC, Gillikin JW, Larkins BA, Boston RS, Jung R 2006 The maize *Mucronate* mutation is a deletion in the 16-kDa γ-zein gene that induces the unfolded protein response. The Plant Journal 48, 440–451. 17010110 10.1111/j.1365-313X.2006.02884.x

[CIT0023] Kim CS, Woo Ym YM, Clore AM, Burnett RJ, Carneiro NP, Larkins BA 2002 Zein protein interactions, rather than the asymmetric distribution of zein mRNAs on endoplasmic reticulum membranes, influence protein body formation in maize endosperm. The Plant Cell 14, 655–672. 11910012 10.1105/tpc.010431 PMC150587

[CIT0024] Klein EM, Mascheroni L, Pompa A, Ragni L, Weimar T, Lilley KS, Dupree P, Vitale A 2006 Plant endoplasmin supports the protein secretory pathway and has a role in proliferating tissues. The Plant Journal 48, 657–673. 17059403 10.1111/j.1365-313X.2006.02904.x

[CIT0025] Kogan MJ, López O, Cocera M, López-Iglesias C, De La Maza A, Giralt E 2004 Exploring the interaction of the surfactant N-terminal domain of γ-Zein with soybean phosphatidylcholine liposomes. Biopolymers 73, 258–268. 14755582 10.1002/bip.10578

[CIT0026] Larkins BA, Hurkman WJ 1978 Synthesis and deposition of zein in protein bodies of maize endosperm. Plant Physiology 62, 256–263. 16660496 10.1104/pp.62.2.256 PMC1092100

[CIT0027] Lending CR, Larkins BA 1989 Changes in the zein composition of protein bodies during maize endosperm development. The Plant Cell 1, 1011–1023. 2562552 10.1105/tpc.1.10.1011 PMC159838

[CIT0028] Llop-Tous I, Madurga S, Giralt E, Marzabal P, Torrent M, Ludevid MD 2010 Relevant elements of a maize γ-zein domain involved in protein body biogenesis. The Journal of Biological Chemistry 285, 35633–35644. 20829359 10.1074/jbc.M110.116285 PMC2975188

[CIT0029] Mainieri D, Morandini F, Maîtrejean M, Saccani A, Pedrazzini E, Alessandro V 2014 Protein body formation in the endoplasmic reticulum as an evolution of storage protein sorting to vacuoles: insights from maize γ-zein. Frontiers in Plant Science 5, 331. 25076952 10.3389/fpls.2014.00331 PMC4097401

[CIT0030] Mainieri D, Rossi M, Archinti M, Bellucci M, De Marchis F, Vavassori S, Pompa A, Arcioni S, Vitale A 2004 Zeolin. A new recombinant storage protein constructed using maize γ-zein and bean phaseolin. Plant Physiology 136, 3447–3456. 15502013 10.1104/pp.104.046409 PMC527144

[CIT0031] Misra PS, Mertz ET, Glover DV 1976 Studies on corn proteins. IX. Comparison of the amino acid composition of Landry-Moureaux and Paulis-Wall endosperm fractions. Cereal Chemistry 53, 699–704.

[CIT0032] Muench DG, Chuong SD, Franceschi VR, Okita TW 2000 Developing prolamine protein bodies are associated with the cortical cytoskeleton in rice endosperm cells. Planta 211, 227–238. 10945217 10.1007/PL00008159

[CIT0033] Pedrazzini E, Mainieri D, Marrano CA, Vitale A 2016 Where do protein bodies of cereal seeds come from?Frontiers in Plant Science 7, 1139. 27540384 10.3389/fpls.2016.01139 PMC4973428

[CIT0034] Phan HT, Hause B, Hause G, Arcalis E, Stoger E, Maresch D, Altmann F, Joensuu J, Conrad U 2014 Influence of elastin-like polypeptide and hydrophobin on recombinant hemagglutinin accumulations in transgenic tobacco plants. PLoS ONE 9, e99347. 24914995 10.1371/journal.pone.0099347 PMC4051685

[CIT0035] Pompa A, Vitale A 2006 Retention of a bean phaseolin/maize γ-Zein fusion in the endoplasmic reticulum depends on disulfide bond formation. The Plant Cell 18, 2608–2621. 17041149 10.1105/tpc.106.042226 PMC1626613

[CIT0036] Prat S, Cortadas J, Puigdomènech P, Palau J 1985 Nucleic acid (cDNA) and amino acid sequences of the maize endosperm protein glutelin-2. Nucleic Acids Research 13, 1493–1504. 3839076 10.1093/nar/13.5.1493 PMC341091

[CIT0037] Prat S, Pérez-Grau L, Puigdomènech P 1987 Multiple variability in the sequence of a family of maize endosperm proteins. Gene 52, 41–49. 3596247 10.1016/0378-1119(87)90393-3

[CIT0038] Reyes FC, Chung T, Holding D, Jung R, Vierstra R, Otegui MS 2011 Delivery of prolamins to the protein storage vacuole in maize aleurone cells. The Plant Cell 23, 769–784. 21343414 10.1105/tpc.110.082156 PMC3077793

[CIT0039] Saito Y, Kishida K, Takata K, Takahashi H, Shimada T, Tanaka K, Morita S, Satoh S, Masumura T 2009 A green fluorescent protein fused to rice prolamin forms protein body-like structures in transgenic rice. Journal of Experimental Botany 60, 615–627. 19129168 10.1093/jxb/ern311 PMC2651459

[CIT0040] Saito Y, Shigemitsu T, Yamasaki R, et al 2012 Formation mechanism of the internal structure of type I protein bodies in rice endosperm: relationship between the localization of prolamin species and the expression of individual genes. The Plant Journal 70, 1043–1055. 22348505 10.1111/j.1365-313X.2012.04947.x

[CIT0041] Salamini F, Di Fonzo N, Fornasari E, Gentinetta E, Reggiani R, Soave C 1983 *Mucronate*, *Mc*, a dominant gene of maize which interacts with *opaque*-2 to suppress zein synthesis. Theoretical and Applied Genetics 65, 123–128. 24263340 10.1007/BF00264879

[CIT0042] Shewry PR, Halford NG 2002 Cereal seed storage proteins: structures, properties and role in grain utilization. Journal of Experimental Botany 53, 947–958. 11912237 10.1093/jexbot/53.370.947

[CIT0043] Shull JM, Watterson JJ, Kirleis AW 1992 Purification and immunocytochemical localization of kafirins in *Sorghum bicolor* (L. Moench) endosperm. Protoplasma 171, 64–74.

[CIT0044] Swigonˇová Z, Lai J, Ma J, Ramakrishna W, Llaca V, Bennetzen JL, Messing J 2004 Close split of sorghum and maize genome progenitors. Genome Research 14, 1916–1923. 15466289 10.1101/gr.2332504 PMC524415

[CIT0045] Torrent M, Llompart B, Lasserre-Ramassamy S, Llop-Tous I, Bastida M, Marzabal P, Westerholm-Parvinen A, Saloheimo M, Heifetz PB, Ludevid MD 2009 Eukaryotic protein production in designed storage organelles. BMC Biology 7, 5. 19175916 10.1186/1741-7007-7-5 PMC2637842

[CIT0046] Vitale A, Smaniotto E, Longhi R, Galante E 1982 Reduced soluble proteins associated with maize endosperm protein bodies. Journal of Experimental Botany 33, 439–448.

[CIT0047] Wang J, Shen J, Cai Y, Robinson DG, Jiang L 2013 Successful transport to the vacuole of heterologously expressed mung bean 8S globulin occurs in seed but not in vegetative tissues. Journal of Experimental Botany 64, 1587–1601. 23382549 10.1093/jxb/ert014 PMC3617825

[CIT0048] Washida H, Sugino A, Messing J, Esen A, Okita TW 2004 Asymmetric localization of seed storage protein RNAs to distinct subdomains of the endoplasmic reticulum in developing maize endosperm cells. Plant & Cell Physiology 45, 1830–1837. 15653801 10.1093/pcp/pch210

[CIT0049] Wong JW, Ho SY, Hogg PJ 2011 Disulfide bond acquisition through eukaryotic protein evolution. Molecular Biology and Evolution 28, 327–334. 20675408 10.1093/molbev/msq194

[CIT0050] Woo YM, Hu DW, Larkins BA, Jung R 2001 Genomics analysis of genes expressed in maize endosperm identifies novel seed proteins and clarifies patterns of zein gene expression. The Plant Cell 13, 2297–2317. 11595803 10.1105/tpc.010240 PMC139160

[CIT0051] Wu Y, Messing J 2010 RNA interference-mediated change in protein body morphology and seed opacity through loss of different zein proteins. Plant Physiology 153, 337–347. 20237020 10.1104/pp.110.154690 PMC2862413

[CIT0052] Xu J-H, Messing J 2008 Organization of the prolamin gene family provides insight into the evolution of the maize genome and gene duplications in grass species. Proceedings of the National Academy of Sciences, USA 105, 14330–14335. 10.1073/pnas.0807026105 PMC2567223 18794528

[CIT0053] Xu J-H, Messing J 2009 Amplification of prolamin storage protein genes in different subfamilies of the Poaceae. Theoretical and Applied Genetics 119, 1397–1412. 19727653 10.1007/s00122-009-1143-x

[CIT0054] Yamagata T, Kato H, Kuroda S, Abe S, Davies E 2003 Uncleaved legumin in developing maize endosperm: identification, accumulation and putative subcellular localization. Journal of Experimental Botany 54, 913–922. 12598562 10.1093/jxb/erg090

[CIT0055] Yao D, Qi W, Li X, Yang Q, Yan S, Ling H, Wang G, Wang G, Song R 2016 Maize *opaque10* encodes a cereal-specific protein that is essential for the proper distribution of zeins in endosperm protein bodies. PLoS Genetics 12, e1006270. 27541862 10.1371/journal.pgen.1006270 PMC4991801

[CIT0056] Yu M, Lau TY, Carr SA, Krieger M 2012 Contributions of a disulfide bond and a reduced cysteine side chain to the intrinsic activity of the high-density lipoprotein receptor SR-BI. Biochemistry 51, 10044–10055. 23205738 10.1021/bi301203x PMC3566775

[CIT0057] Zhang F, Boston RS 1992 Increases in binding protein (BiP) accompany changes in protein body morphology in three high-lysine mutants of maize. Protoplasma 171, 142–152.

