# Supplementary material for: Maize 16-kD γ-zein forms very unusual disulfide-bonded polymers in the endoplasmic reticulum: implications for prolamin evolution
Source: J Exp Bot. 2018 Aug 2;69(21):5013–27. doi: 10.1093/jxb/ery287 (PMC6184761; doi:10.1093/jxb/ery287)
Supplement: Supplementary Materials [file ery287_suppl_supplementary_materials.pdf]

Supplementary Data

Supplementary Protocol S1 (associated with Fig. S1)

Protein in-gel digestion and LC-ESI-MS/MS analysis

Considering that chymotrypsin digestion is the treatment of choice for zeins but it could mask the presence of other proteins (Sergeant et al., 2009), the MS analysis of each band was done both after chymotrypsin and after trypsin digestion. The in-gel digestion of each band (see Supplemental Figure S1) was done as previously described (Prinsi et al., 2016) with the following refinements. For the cysteine reduction and alkylation steps, the samples were incubated in 25 mM DTT, 100 mM NH_4HCO_3 for 45 min at 56°C. The solution was replaced with 110 mM iodoacetamide, 100 mM NH_4HCO_3 and the bands were incubated for 30 min in the dark at room temperature. For the protein digestion enzyme solution was added to each sample. Chymotrypsin (V1060, Promega) was dissolved in 30 mM NH_4HCO_3, 10 mM $CaCl_2$, pH 8.0. Trypsin (V5111, Promega) was dissolved in 25 mM NH_4HCO_3, 2.5 mM $CaCl_2$, pH 7.8. After incubation for 45 min at 4 °C, the supernatants were replaced with digestion buffer without protease and the samples were incubated for 16 h at 25°C or 37 °C for chymotrypsin or trypsin digestion, respectively. Afterwards, recovery of peptides and LC-ESI-MS/MS analysis by a 6520 Q-TOF (Agilent Technologies) was done as previously described (Prinsi et al., 2016).

Peptide identification was performed by Spectrum Mill MS Proteomics Workbench (Rev B.04.00.127; Agilent Technologies). Cysteine carbamidomethylation and methionine oxidation were set as fixed and variable modifications, respectively, accepting two missed cleavages *per* peptide. The search was conducted against the subset of *Zea mays* protein sequences downloaded from the National Center for Biotechnology Information (http://www.ncbi.nlm.nih.gov/) and concatenated with the reverse one. The thresholds for peptide validation were False Discovery Rate < 1%, Database Fwd-Rev Score ≥ 2, Score Peak Intensity ≥ 70% and mass MH^+ Error < ±10 ppm. Protein identification was accepted only if supported by at least two peptides covering the 10% of the sequence. Each sample was analyzed twice. For each gel band, the purity of the most abundant protein was estimated as its protein spectrum intensity percentage (%PSI, % of all the proteins in the band).

References

Prinsi B, Negri AS, Quattrocchio FM, Koes RE, Espen L. 2016. Proteomics of red and white corolla limbs in petunia reveals a novel function of the anthocyanin regulator ANTHOCYANIN1 in determining flower longevity. Journal of Proteomics **131,** 38-47.

Sergeant K, Pinhero C, Hausman J-F, Ricardo CP, Renaut J. 2009. Taking advantage of nonspecific trypsin cleavages for the identification of seed storage proteins in cereals. J Proteome Research **8,** 3182-3190.

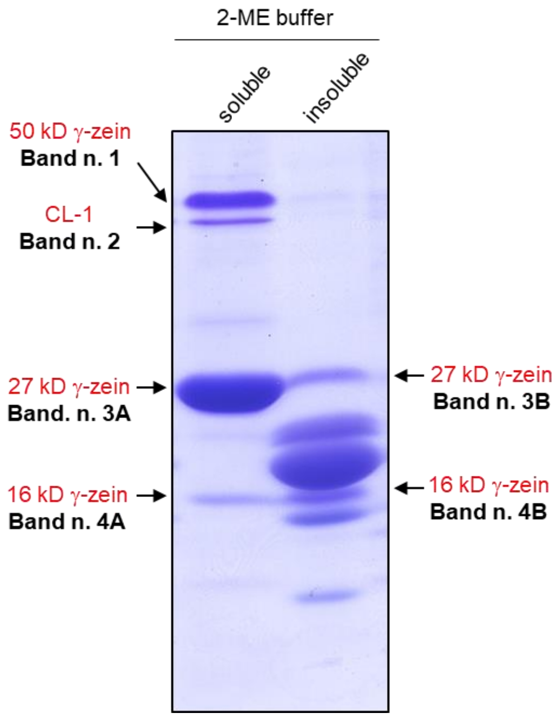

GEL BAND n. 1

>NP_001105053.2

50 kDa gamma-zein precursor [Zea mays]

MKLVLVVLAFIALVSSVSCTQTGGCSCGQQQSHEQQHHPQQHHPQKQQHQPPPQHHQQQHQQQV
HMQPQKHQQQQEVHVQQQQQQPQHQQQQQQQHQQQHQCEGQQQHHQQSQGHVQQHEQSHEQHQ
GQSHEQQHQQQFQGHDKQQQPQQPQQYQQGQEKSQQQQCHCQEQQQTTRCSYNYYSSSSNLKNC
HEFLRQQCSPLVMPFLQSRLIQPSSCQVLQQQCCHDLRQIEPQYIHQAIYNMVQSIIQEEQQQQPCE
LCGSQQATQSAVAILTAAQYLPSMCGLYHSYYQNNPCSSNDISGVCN

GEL BAND n. 2

>NP_001104865.2

legumin 1 precursor [Zea mays]

MAAAIVLSGQVRPLPSSLPLSLLLLLLLCCSGTSWGWSTSRGGAARECGFDGKLEALEPRHKVQSEAG
SVQYFSRFNEADRELTCAGIFAVRVVVDAMGLLLPRYSNVHSLVYIVQGRGIIGFSFPGCQEETQQQ
QYGYGYGYGHHHHQHDHHKIHRFEQGDVVAMPAGAQHWLYNDGDAPLVAVYVFDENNNINQLEPS
MRKFLLAGGFSKGQPHFAENIFKGIDARFLSEALGVSMHVAEKLQSRRDQRGEIVRVEPEHGFHQL
NPSPSSSSFSFPSSQVQYQTCQRDVDRHNVCAMEVRHSVERLDQADVYSPGAGRITRLTSHKFPVLNL
VQMSAVRVDLYQDAIMSPFWNFNAHSAMYGIRGSARVQVASDNGTTVFDDVLRAGQLLIVPQGYL
VATKAQGEGFQYIAFETNPDTMVSHVAGKNSVLSDLPAAVIASSYAISMEEAAELKNGRKHELAVL
TPAGSGSYQQGQAGSAQQ

GEL BAND n. 3A

>sp|P04706.1|

GLU2_MAIZE RecName: Full=Glutelin-2; AltName: Full=27 kDa zein; AltName: Full=Alcohol-soluble reduced glutelin; Short=ASG; AltName: Full=Zein Zc2; AltName: Full=Zein-gamma; Flags: Precursor

MRVLLVALALLALAASATSTHTSGGCGCQPPPPVHLPPPVHLPPPVHLPPPVHLPPPVHLPPPVHLPPPV
HVPPPVHLPPPPCHYPTQPPRPQPHPQPHPCPCQQPHPSPCQL**QGTCGVGSTPILGQCVEFL**RHQCSPT
ATPYCSPQCQSLR**QQCCQQLRQVEPQHR**YQAIFGLVL**QSILQQQPQSGQVAGLLAAQIAQQLTAM**
CGLQQPTPCPYAAAGGVPH

GEL BAND n. 3B

>sp|P04706.1|

GLU2_MAIZE RecName: Full=Glutelin-2; AltName: Full=27 kDa zein; AltName: Full=Alcohol-soluble reduced glutelin; Short=ASG; AltName: Full=Zein Zc2; AltName: Full=Zein-gamma; Flags: Precursor

MRVLLVALALLALAASATSTHTSGGCGCQPPPPVHLPPPVHLPPPVHLPPPVHLPPPVHLPPPVHLPPPV
HVPPPVHLPPPPCHYPTQPPRPQPHPQPHPCPCQQPHPSPCQL**QGTCGVGSTPILGQCVEFLR**HQCSPT
ATPYCSPQCQSLR**QQCCQQLR**QVEPQHRYQAIFGLVL**QSILQQQPQSGQVAGLLAAQIAQQLTAMC**
GLQQPTPCPYAAAGGVPH

GEL BAND n. 4A

>sp|P08031.1|

ZEB2_MAIZE RecName: Full=16 kDa gamma-zein; AltName: Full=16 kDa zein; AltName: Full=Zein Zc1; AltName: Full=Zein-2; AltName: Full=Zein-gamma; Flags: Precursor

MKVLIVALALLALAASAASSTSGGCGCQTPPFHLPPPFYMPPPF**YLPPQQQPQPWQYPTQPPQLSPCQ**
QFGSCGVGSVGSPFLGQCVEF**LRHQCSPAATPYGSPQCQAL**QQQCCHQIR**QVEPLHR**YQATY**GVVL**
QSFLQQQPQGELAALMAAQVAQQLTAMCGL**QLQQPGPCPCNAAAGGVY**Y

GEL BAND n. 4B

>sp|P08031.1|

ZEB2_MAIZE RecName: Full=16 kDa gamma-zein; AltName: Full=16 kDa zein; AltName: Full=Zein Zc1; AltName: Full=Zein-2; AltName: Full=Zein-gamma; Flags: Precursor

MKVLIVALALLALAASAASSTSGGCGCQTPPF**HLPPPFYMPPPFYLPPQQQPQPWQYPTQPPQLSPC**
QQFGSCGVGSVGSPFLGQCVEFLRHQCSPAATPYGSPQCQALQQQCCHQIR**QVEPLHR**YQATYGVV
L**QSFLQQQPQGELAALMAAQVAQQLTAMCGLQLQQPGPCPCNAAAGGVYY**

Fig. S1. Identities of the major polypeptides present in purified maize PBs, as determined by LC-ESI-MS/MS analysis. Protein bodies purified from maize seeds, at 25 DAP, were treated at 4°C with buffer containing 4% 2-ME (see Figure 2A). The numbering of each gel band used for mass spectrometry identification is reported near the corresponding band in the stained SDS-PAGE gel. In each protein sequence identified by mass spectrometry, the peptides identified after chymotrypsin (red) or trypsin (underscored) in-gel digestion are indicated. For further MS/MS data see Supplementry Table S1.

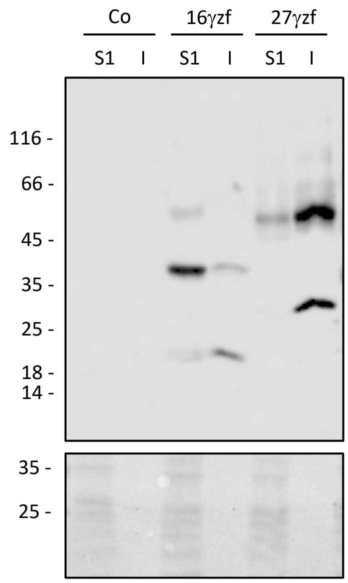

Fig. S2. Variability in denaturation-resistant oligomers. Protoplasts were isolated from tobacco leaves and transiently transformed with plasmid encoding 27γzf or 16γzf. After incubation for 20 h, protoplasts were homogenized in the absence of 2-ME. After centrifugation, soluble (S1) and insoluble (I) fractions were collected. Proteins in each fraction were analyzed by SDS-PAGE and protein blot with anti-FLAG antibody. Top image is protein blot, bottom image is Ponceau S staining. Numbers at left indicate the positions of SDS-PAGE molecular mass markers, in kD. This is a fully independent repetition of the experiment shown in Fig. 3A, with the only difference that in this case the material insoluble in non-reducing conditions was directly analyzed; therefore, fraction I contains also the proteins in fraction S2 of Fig. 3A. Notice that, as in Fig. 3A, 16γzf is more soluble that 27γzf in non-reducing conditions, but in this case much higher proportions of both 27γzf and 16γzf oligomers remain assembled, in spite of the denaturation treatment for SDS-PAGE.

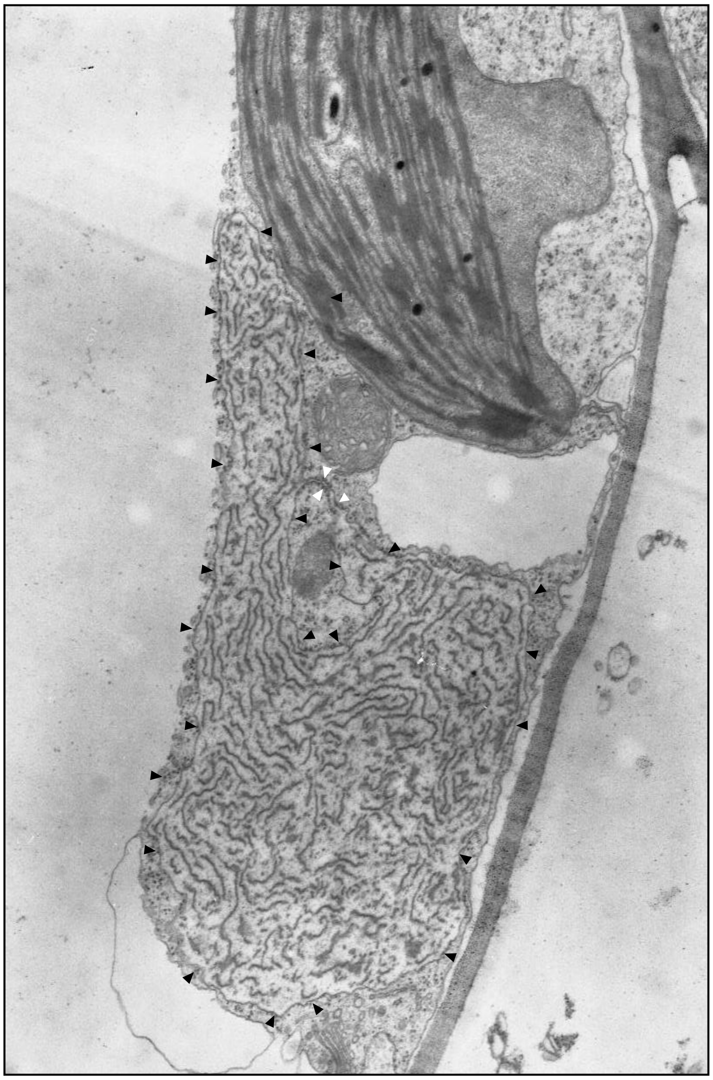

Fig. S3. Dilated ER in leaf cells of transgenic Arabidopsis expressing 16γzf. This is the same image shown in Fig. 7A. The black arrowheads follow the perimeter of the enlarged ER, pointing from the cytosol towards the ER membrane. The white arrowheads point, from the same direction, to tubular ER connected to the dilated structure.

Supplemental Table 1. Peptide identification by LC-ESI-MS/MS analysis, and protein assignment. SDS-PAGE band numbers are as in Supplemental Figure 1.
PS: Protein Score; **PSI**: protein spectrum intensity (sum of all the peptide spectrum intensities assigned to the protein); **%PSI**: PSI as percentage of the sum of all the proteins in the band;
E: protease used for digestion (**c:** chymotrypsin**; t:** trypsin)**; DS**: discriminant score

<table>
<tr><th>Band</th><th>Protein Accession Number</th><th>petide number: Unique (Total)</th><th>aa coverage (%)</th><th>PS</th><th>PSI</th><th>%PSI</th><th>E</th><th>z</th><th>Score</th><th>DS</th><th>FDR</th><th>Database Fwd-Rev Score</th><th>Score Peak Intensity (%)</th><th>Spectrum Intensity</th><th>Start AA position</th><th>Sequence map for distinct peptides (forward-slashes for locations of y-ions; backslashes for locations of b-ions; vertical lines for locations of both b- and y-ions; m: oxidized methionine)</th><th>precursor m/z measured (Da)</th><th>precursor MH+ matched (Da)</th><th>MH+ error (ppm)</th></tr>
<tr><td>1</td><td>NP_001105053.2</td><td>18 (34)</td><td>46%</td><td>282,41</td><td>1,35E+08</td><td>96%</td><td>c</td><td>2</td><td>15,21</td><td>16,804</td><td><0.1%</td><td>11,72</td><td>91,70</td><td>6,69E+06</td><td>143</td><td>(F)Q G/H D\K Q Q Q|P Q|Q|P/Q|Q\Y(Q)</td><td>919,4328</td><td>1837,858</td><td>0,4</td></tr>
<tr><td></td><td></td><td></td><td></td><td></td><td></td><td></td><td>t</td><td>2</td><td>22,22</td><td>21,621</td><td><0.1%</td><td>22,22</td><td>90,80</td><td>7,96E+06</td><td>148</td><td>(K)Q Q|Q|P Q|Q|P Q/Q/Y/Q/Q/G Q/E K(S)</td><td>985,9605</td><td>1970,931</td><td>-9</td></tr>
<tr><td></td><td></td><td></td><td></td><td></td><td></td><td></td><td>t</td><td>2</td><td>22</td><td>22,394</td><td><0.1%</td><td>22,00</td><td>95,70</td><td>1,78E+07</td><td>180</td><td>(R)C S|Y|N|Y|Y|S/S/S/S/N L/K(N)</td><td>786,8319</td><td>1572,664</td><td>-4,4</td></tr>
<tr><td></td><td></td><td></td><td></td><td></td><td></td><td></td><td>c</td><td>2</td><td>17,24</td><td>15,252</td><td><0.1%</td><td>12,46</td><td>92,70</td><td>3,85E+06</td><td>186</td><td>(Y)S/S|S/S N/L/K N C|H\E\F(L)</td><td>705,3126</td><td>1409,611</td><td>4,6</td></tr>
<tr><td></td><td></td><td></td><td></td><td></td><td></td><td></td><td>c</td><td>2</td><td>17,63</td><td>16,111</td><td><0.1%</td><td>11,51</td><td>98,10</td><td>7,85E+05</td><td>186</td><td>(Y)S S|S/S N L/K N|C\H\E|F\L(R)</td><td>761,8515</td><td>1522,696</td><td>0,2</td></tr>
<tr><td></td><td></td><td></td><td></td><td></td><td></td><td></td><td>c</td><td>2</td><td>14,88</td><td>12,155</td><td><0.1%</td><td>9,03</td><td>93,20</td><td>1,50E+06</td><td>192</td><td>(L)K|N/C/H\E|F(L)</td><td>417,6838</td><td>834,356</td><td>4,8</td></tr>
<tr><td></td><td></td><td></td><td></td><td></td><td></td><td></td><td>t</td><td>2</td><td>12,7</td><td>13,755</td><td><0.1%</td><td>12,70</td><td>70,80</td><td>1,98E+07</td><td>193</td><td>(K)N|C|H/E|F/L/R(Q)</td><td>488,2234</td><td>975,447</td><td>-7,2</td></tr>
<tr><td></td><td></td><td></td><td></td><td></td><td></td><td></td><td>t</td><td>2</td><td>19,94</td><td>19,047</td><td><0.1%</td><td>19,94</td><td>94,60</td><td>1,40E+07</td><td>200</td><td>(R)Q Q|C/S/P L/V|m/P F/L/Q/S R(L)</td><td>853,9146</td><td>1690,840</td><td>-7,8</td></tr>
<tr><td></td><td></td><td></td><td></td><td></td><td></td><td></td><td>c</td><td>2</td><td>12,97</td><td>15,387</td><td><0.1%</td><td>7,76</td><td>92,00</td><td>7,82E+06</td><td>210</td><td>(F)L Q\S R\L I|Q|P S\S\C\Q|V\L(Q)</td><td>814,9464</td><td>1628,879</td><td>4,1</td></tr>
<tr><td></td><td></td><td></td><td></td><td></td><td></td><td></td><td>c</td><td>2</td><td>11,9</td><td>12,879</td><td><0.1%</td><td>7,09</td><td>93,10</td><td>3,72E+05</td><td>211</td><td>(L)Q S R L I|Q|P S\S\C\Q|V\L(Q)</td><td>758,4050</td><td>1515,795</td><td>5,2</td></tr>
<tr><td></td><td></td><td></td><td></td><td></td><td></td><td></td><td>c</td><td>2</td><td>15,54</td><td>17,324</td><td><0.1%</td><td>15,54</td><td>95,00</td><td>9,58E+05</td><td>232</td><td>(L)R Q I/E|P Q\Y|I|H|Q\A\I\Y(N)</td><td>829,9406</td><td>1658,865</td><td>5,4</td></tr>
<tr><td></td><td></td><td></td><td></td><td></td><td></td><td></td><td>c</td><td>2</td><td>18,31</td><td>20,686</td><td><0.1%</td><td>18,31</td><td>94,40</td><td>4,17E+05</td><td>245</td><td>(Y)N M\V\Q\S I|I|Q E\E Q/Q/Q|Q|P/C E L(C)</td><td>1101,5145</td><td>2202,017</td><td>2,4</td></tr>
<tr><td></td><td></td><td></td><td></td><td></td><td></td><td></td><td>c</td><td>2</td><td>8,81</td><td>13,022</td><td><0.1%</td><td>2,48</td><td>76,10</td><td>5,94E+06</td><td>263</td><td>(L)C G S Q Q A\T Q\S\A\V|A|I\L(T)</td><td>717,3595</td><td>1433,705</td><td>4,5</td></tr>
<tr><td></td><td></td><td></td><td></td><td></td><td></td><td></td><td>c</td><td>2</td><td>11,41</td><td>11,697</td><td><0.1%</td><td>11,41</td><td>89,50</td><td>1,27E+05</td><td>277</td><td>(L)T A\A Q\Y|L/P S M/C/G/L(Y)</td><td>656,3094</td><td>1311,607</td><td>3,3</td></tr>
<tr><td></td><td></td><td></td><td></td><td></td><td></td><td></td><td>c</td><td>1</td><td>12,78</td><td>15,96</td><td><0.1%</td><td>9,68</td><td>90,60</td><td>1,22E+05</td><td>277</td><td>(L)T A A|Q|Y|L/P\S M\C\G\L\Y(H)</td><td>1474,6720</td><td>1474,670</td><td>1</td></tr>
<tr><td></td><td></td><td></td><td></td><td></td><td></td><td></td><td>c</td><td>2</td><td>15,68</td><td>15,46</td><td><0.1%</td><td>15,68</td><td>87,40</td><td>3,64E+04</td><td>290</td><td>(Y)H S Y Y\Q\N N|P C S S N D/I|S G\V|C N(-)</td><td>1108,4413</td><td>2215,877</td><td>-0,6</td></tr>
<tr><td></td><td></td><td></td><td></td><td></td><td></td><td></td><td>c</td><td>2</td><td>18,04</td><td>19,86</td><td><0.1%</td><td>18,04</td><td>93,50</td><td>5,88E+05</td><td>293</td><td>(Y)Y Q\N N|P C\S\S N D\I|S|G/V|C/N(-)</td><td>914,8721</td><td>1828,723</td><td>7,9</td></tr>
<tr><td></td><td></td><td></td><td></td><td></td><td></td><td></td><td>c</td><td>2</td><td>15,15</td><td>16,69</td><td><0.1%</td><td>15,15</td><td>88,50</td><td>3,38E+06</td><td>294</td><td>(Y)Q N\N|P C\S S N D\I|S|G/V|C N(-)</td><td>833,3373</td><td>1665,659</td><td>4,9</td></tr>
<tr><td>2</td><td>NP_001104865.2</td><td>30 (66)</td><td>51%</td><td>520,46</td><td>2,06E+08</td><td>99%</td><td>t</td><td>2</td><td>15,92</td><td>17,80</td><td><0.1%</td><td>15,92</td><td>71,40</td><td>1,48E+05</td><td>47</td><td>(R)E/C/G F/D/G K/L/E/A/L/E|P R(H)</td><td>810,8919</td><td>1620,769</td><td>4,9</td></tr>
<tr><td></td><td></td><td></td><td></td><td></td><td></td><td></td><td>t</td><td>2</td><td>24,7</td><td>24,30</td><td><0.1%</td><td>20,28</td><td>93,90</td><td>1,14E+07</td><td>63</td><td>(K)V/Q|S|E|A|G|S/V/Q|Y/F/S/R(F)</td><td>729,3562</td><td>1457,702</td><td>2,2</td></tr>
<tr><td></td><td></td><td></td><td></td><td></td><td></td><td></td><td>c</td><td>2</td><td>12,45</td><td>12,76</td><td><0.1%</td><td>4,12</td><td>93,90</td><td>3,64E+06</td><td>77</td><td>(F)N E\A D R E L\T|C\A\G|I|F(A)</td><td>748,3475</td><td>1495,685</td><td>2,1</td></tr>
<tr><td></td><td></td><td></td><td></td><td></td><td></td><td></td><td>c</td><td>2</td><td>15,39</td><td>14,78</td><td><0.1%</td><td>8,05</td><td>95,70</td><td>2,70E+05</td><td>90</td><td>(F)A V R\V V\V|D\A|m|G|L(L)</td><td>573,3227</td><td>1129,640</td><td>3,0</td></tr>
<tr><td></td><td></td><td></td><td></td><td></td><td></td><td></td><td>t</td><td>2</td><td>22,9</td><td>20,79</td><td><0.1%</td><td>15,90</td><td>94,80</td><td>3,63E+06</td><td>93</td><td>(R)V V|V|D|A|m/G/L|L|L|P R(Y)</td><td>649,8780</td><td>1282,755</td><td>-1,0</td></tr>
<tr><td></td><td></td><td></td><td></td><td></td><td></td><td></td><td>t</td><td>2</td><td>26,08</td><td>26,62</td><td><0.1%</td><td>26,08</td><td>100,00</td><td>2,78E+06</td><td>105</td><td>(R)Y/S/N|V|H|S|L/V|Y|I/V/Q/G R(G)</td><td>817,9377</td><td>1634,865</td><td>2,0</td></tr>
<tr><td></td><td></td><td></td><td></td><td></td><td></td><td></td><td>c</td><td>2</td><td>18,22</td><td>13,78</td><td><0.1%</td><td>9,43</td><td>95,30</td><td>1,35E+07</td><td>114</td><td>(Y)I/V|Q|G R G I|I|G|F(S)</td><td>530,3222</td><td>1059,631</td><td>5,8</td></tr>
<tr><td></td><td></td><td></td><td></td><td></td><td></td><td></td><td>c</td><td>2</td><td>17,35</td><td>18,27</td><td><0.1%</td><td>17,35</td><td>96,50</td><td>2,58E+06</td><td>159</td><td>(F)E Q|G D/V|V|A|M|P A/G A Q/H W(L)</td><td>798,3710</td><td>1595,727</td><td>4,8</td></tr>
<tr><td></td><td></td><td></td><td></td><td></td><td></td><td></td><td>t</td><td>2</td><td>16,3</td><td>16,90</td><td><0.1%</td><td>13,26</td><td>89,00</td><td>1,28E+06</td><td>203</td><td>(R)K|F|L|L|A/G G F|S|K(G)</td><td>534,3190</td><td>1067,625</td><td>5,6</td></tr>
<tr><td></td><td></td><td></td><td></td><td></td><td></td><td></td><td>c</td><td>2</td><td>14,44</td><td>15,01</td><td><0.1%</td><td>6,99</td><td>83,40</td><td>2,27E+06</td><td>211</td><td>(F)S K|G Q|P H\F\A\E\N|I|F(K)</td><td>687,8480</td><td>1374,680</td><td>6,3</td></tr>
<tr><td></td><td></td><td></td><td></td><td></td><td></td><td></td><td>t</td><td>2</td><td>19,03</td><td>15,35</td><td><0.1%</td><td>9,57</td><td>91,40</td><td>5,00E+06</td><td>213</td><td>(K)G Q|P/H|F|A E/N I|F/K(G)</td><td>644,3300</td><td>1287,648</td><td>3,6</td></tr>
<tr><td></td><td></td><td></td><td></td><td></td><td></td><td></td><td>c</td><td>2</td><td>14,07</td><td>11,64</td><td><0.1%</td><td>6,72</td><td>89,20</td><td>2,27E+06</td><td>223</td><td>(F)K|G|I|D|A R/F\L(S)</td><td>460,2731</td><td>919,536</td><td>3,2</td></tr>
<tr><td></td><td></td><td></td><td></td><td></td><td></td><td></td><td>c</td><td>2</td><td>13,08</td><td>14,77</td><td><0.1%</td><td>7,20</td><td>91,70</td><td>1,89E+05</td><td>223</td><td>(F)K|G I/D|A\R F\L\S\E|A|L(G)</td><td>660,3729</td><td>1319,732</td><td>5,1</td></tr>
<tr><td></td><td></td><td></td><td></td><td></td><td></td><td></td><td>t</td><td>2</td><td>23,21</td><td>21,30</td><td><0.1%</td><td>23,21</td><td>87,20</td><td>1,44E+06</td><td>229</td><td>(R)F L|S/E/A|L|G/V S m H/V|A E/K(L)</td><td>817,4199</td><td>1617,831</td><td>4,3</td></tr>
<tr><td></td><td></td><td></td><td></td><td></td><td></td><td></td><td>c</td><td>2</td><td>18,79</td><td>19,44</td><td><0.1%</td><td>18,79</td><td>96,00</td><td>1,47E+05</td><td>230</td><td>(F)L/S/E/A|L|G V/S m H V|A E|K\L(Q)</td><td>800,4247</td><td>1583,846</td><td>0,7</td></tr>
<tr><td></td><td></td><td></td><td></td><td></td><td></td><td></td><td>c</td><td>2</td><td>13,22</td><td>12,39</td><td><0.1%</td><td>13,22</td><td>73,40</td><td>6,30E+04</td><td>235</td><td>(L)G V|S M/H\V A\E|K L(Q)</td><td>535,7891</td><td>1070,566</td><td>4,3</td></tr>
<tr><td></td><td></td><td></td><td></td><td></td><td></td><td></td><td>t</td><td>2</td><td>15,31</td><td>16,93</td><td><0.1%</td><td>15,31</td><td>84,60</td><td>5,91E+06</td><td>294</td><td>(R)H|N|V|C/A|M\E|V/R(H)</td><td>558,2598</td><td>1115,508</td><td>3,5</td></tr>
<tr><td></td><td></td><td></td><td></td><td></td><td></td><td></td><td>t</td><td>2</td><td>19,65</td><td>20,03</td><td><0.1%</td><td>19,65</td><td>98,00</td><td>1,39E+07</td><td>308</td><td>(R)L D|Q|A|D|V|Y|S|P G A/G R(I)</td><td>674,8311</td><td>1348,649</td><td>4,3</td></tr>
<tr><td></td><td></td><td></td><td></td><td></td><td></td><td></td><td>c</td><td>2</td><td>11,07</td><td>12,64</td><td><0.1%</td><td>5,85</td><td>72,00</td><td>1,04E+05</td><td>325</td><td>(L)T/S/H/K\F\P V\L|N/L(V)</td><td>578,3296</td><td>1155,652</td><td>-0,1</td></tr>
<tr><td></td><td></td><td></td><td></td><td></td><td></td><td></td><td>t</td><td>2</td><td>22,45</td><td>22,77</td><td><0.1%</td><td>22,45</td><td>100,00</td><td>2,33E+06</td><td>329</td><td>(K)F/P|V|L|N|L|V/Q/M/S A V/R(V)</td><td>737,4156</td><td>1473,825</td><td>-0,5</td></tr>
<tr><td></td><td></td><td></td><td></td><td></td><td></td><td></td><td>c</td><td>2</td><td>14,13</td><td>13,42</td><td><0.1%</td><td>14,13</td><td>91,00</td><td>1,56E+05</td><td>354</td><td>(F)W/N|F/N A H S A/m|Y(G)</td><td>628,7622</td><td>1240,520</td><td>1,4</td></tr>
<tr><td></td><td></td><td></td><td></td><td></td><td></td><td></td><td>c</td><td>2</td><td>17,98</td><td>14,50</td><td><0.1%</td><td>11,26</td><td>95,60</td><td>7,21E+05</td><td>383</td><td>(F)D D|V|L/R\A G Q|L|L(I)</td><td>550,3107</td><td>1099,611</td><td>3,2</td></tr>
<tr><td></td><td></td><td></td><td></td><td></td><td></td><td></td><td>t</td><td>2</td><td>16,63</td><td>17,01</td><td><0.1%</td><td>16,63</td><td>97,20</td><td>1,12E+07</td><td>388</td><td>(R)A G\Q\L|L|I|V|P Q G Y L V/A T K(A)</td><td>835,9983</td><td>1670,984</td><td>3,2</td></tr>
<tr><td></td><td></td><td></td><td></td><td></td><td></td><td></td><td>c</td><td>2</td><td>21,38</td><td>17,80</td><td><0.1%</td><td>11,74</td><td>98,10</td><td>3,77E+06</td><td>399</td><td>(Y)L/V|A|T|K/A/Q|G\E|G/F(Q)</td><td>560,8067</td><td>1120,600</td><td>5,7</td></tr>
<tr><td></td><td></td><td></td><td></td><td></td><td></td><td></td><td>c</td><td>2</td><td>14,24</td><td>15,35</td><td><0.1%</td><td>10,99</td><td>88,80</td><td>5,79E+06</td><td>399</td><td>(Y)L V|A|T\K\A\Q\G\E\G F|Q\Y(I)</td><td>706,3609</td><td>1411,722</td><td>-5,0</td></tr>
<tr><td></td><td></td><td></td><td></td><td></td><td></td><td></td><td>c</td><td>2</td><td>18,85</td><td>15,89</td><td><0.1%</td><td>12,91</td><td>95,00</td><td>3,76E+05</td><td>400</td><td>(L)V/A|T/K A/Q|G E|G|F(Q)</td><td>504,2643</td><td>1007,516</td><td>5,6</td></tr>
<tr><td></td><td></td><td></td><td></td><td></td><td></td><td></td><td>c</td><td>2</td><td>16,34</td><td>14,58</td><td><0.1%</td><td>10,05</td><td>90,80</td><td>6,90E+05</td><td>400</td><td>(L)V A|T K A Q|G E\G\F|Q|Y(I)</td><td>649,8236</td><td>1298,638</td><td>1,8</td></tr>
<tr><td></td><td></td><td></td><td></td><td></td><td></td><td></td><td>c</td><td>3</td><td>15,92</td><td>12,74</td><td><0.1%</td><td>9,27</td><td>79,50</td><td>1,20E+05</td><td>415</td><td>(F)E T/N/P/D/T m V S H V/A G K N S\V/L(S)</td><td>638,9795</td><td>1898,928</td><td>0,7</td></tr>
<tr><td></td><td></td><td></td><td></td><td></td><td></td><td></td><td>c</td><td>2</td><td>18,28</td><td>17,51</td><td><0.1%</td><td>18,28</td><td>93,60</td><td>1,07E+06</td><td>433</td><td>(L)S D\L|P A A|V|I|A/S/S/Y(A)</td><td>597,3015</td><td>1193,605</td><td>-7,6</td></tr>
<tr><td></td><td></td><td></td><td></td><td></td><td></td><td></td><td>c</td><td>2</td><td>13,08</td><td>11,62</td><td><0.1%</td><td>2,37</td><td>96,70</td><td>2,39E+06</td><td>445</td><td>(Y)A I\S M/E E\A\A|E|L(K)</td><td>532,2547</td><td>1063,498</td><td>4,3</td></tr>
</table>

<table>
<tr><th>Band</th><th>Protein Accession Number</th><th>petide number: Unique (Total)</th><th>aa coverage (%)</th><th>PS</th><th>PSI</th><th>%PSI</th><th>E</th><th>z</th><th>Score</th><th>DS</th><th>FDR</th><th>Database Fwd-Rev Score</th><th>Score Peak Intensity (%)</th><th>Spectrum Intensity</th><th>Start AA position</th><th>Sequence map for distinct peptides (forward-slashes for locations of y-ions; backslashes for locations of b-ions; vertical lines for locations of both b- and y-ions; m: oxidized methionine)</th><th>precursor m/z measured (Da)</th><th>precursor MH^+ matched (Da)</th><th>MH^+ error (ppm)</th></tr>
<tr><td>3A</td><td>P04706.1</td><td>9 (17)</td><td>32%</td><td>144,28</td><td>1,20E+08</td><td>100%</td><td>c</td><td>2</td><td>17,35</td><td>18,35</td><td><0.1%</td><td>17,35</td><td>90,80</td><td>3,58E+06</td><td>114</td><td>(L)Q G T C G V\G S T|P I|L|G Q|C\V/E/F/L(R)</td><td>1011,9862</td><td>2022,962</td><td>1,4</td></tr>
<tr><td></td><td></td><td></td><td></td><td></td><td></td><td></td><td>t</td><td>2</td><td>15,36</td><td>14,54</td><td><0.1%</td><td>15,36</td><td>91,70</td><td>5,98E+06</td><td>153</td><td>(R)Q|Q|C/C Q/Q/L/R(Q)</td><td>560,7573</td><td>1120,499</td><td>7,8</td></tr>
<tr><td></td><td></td><td></td><td></td><td></td><td></td><td></td><td>t</td><td>2</td><td>14,74</td><td>11,82</td><td><0.1%</td><td>6,16</td><td>79,40</td><td>1,45E+05</td><td>161</td><td>(R)Q/V|E|P Q/H/R(Y)</td><td>447,2362</td><td>893,459</td><td>7,1</td></tr>
<tr><td></td><td></td><td></td><td></td><td></td><td></td><td></td><td>c</td><td>2</td><td>17,58</td><td>18,15</td><td><0.1%</td><td>11,60</td><td>97,50</td><td>2,63E+06</td><td>177</td><td>(L)Q S I\L\Q\Q|Q|P Q\S G Q|V|A/G L(L)</td><td>841,4494</td><td>1681,887</td><td>2,8</td></tr>
<tr><td></td><td></td><td></td><td></td><td></td><td></td><td></td><td>c</td><td>2</td><td>14,33</td><td>17,02</td><td><0.1%</td><td>8,98</td><td>91,30</td><td>6,69E+06</td><td>177</td><td>(L)Q S I\L\Q\Q\Q|P Q S G Q\V\A\G|L L(A)</td><td>897,9979</td><td>1794,971</td><td>9,9</td></tr>
<tr><td></td><td></td><td></td><td></td><td></td><td></td><td></td><td>c</td><td>2</td><td>12,08</td><td>14,25</td><td><0.1%</td><td>7,11</td><td>74,10</td><td>1,24E+06</td><td>181</td><td>(L)Q\Q Q|P\Q\S G Q|V|A|G|L(L)</td><td>620,8197</td><td>1240,628</td><td>3,3</td></tr>
<tr><td></td><td></td><td></td><td></td><td></td><td></td><td></td><td>c</td><td>2</td><td>14,8</td><td>15,09</td><td><0.1%</td><td>9,61</td><td>91,30</td><td>2,28E+07</td><td>181</td><td>(L)Q Q Q|P\Q\S G Q\V|A|G|L L(A)</td><td>677,3612</td><td>1353,712</td><td>2,2</td></tr>
<tr><td></td><td></td><td></td><td></td><td></td><td></td><td></td><td>c</td><td>2</td><td>20,88</td><td>26,12</td><td><0.1%</td><td>20,88</td><td>97,70</td><td>5,79E+05</td><td>194</td><td>(L)A A Q I\A\Q\Q\L\T|A|m|C/G L\Q|Q|P T|P/C/P Y(A)</td><td>1232,0791</td><td>2447,152</td><td>1,8</td></tr>
<tr><td></td><td></td><td></td><td></td><td></td><td></td><td></td><td>c</td><td>2</td><td>17,16</td><td>15,90</td><td><0.1%</td><td>13,92</td><td>98,80</td><td>1,39E+07</td><td>202</td><td>(L)T A\M C G L|Q|Q|P T|P/C|P Y(A)</td><td>812,3546</td><td>1623,696</td><td>3,4</td></tr>
<tr><td>3B</td><td>P04706.1</td><td>10 (19)</td><td>33%</td><td>152,26</td><td>4,93E+07</td><td>84%</td><td>c</td><td>2</td><td>14,6</td><td>15,24</td><td><0.1%</td><td>14,60</td><td>86,90</td><td>2,65E+06</td><td>114</td><td>(L)Q G T C G V\G S T|P I|L|G Q|C\V/E F(L)</td><td>955,4472</td><td>1909,878</td><td>4,6</td></tr>
<tr><td></td><td></td><td></td><td></td><td></td><td></td><td></td><td>c</td><td>2</td><td>16,36</td><td>17,75</td><td><0.1%</td><td>16,36</td><td>82,20</td><td>1,04E+05</td><td>114</td><td>(L)Q G T C G V\G S T|P I|L|G Q/C\V\E|F/L(R)</td><td>1011,9844</td><td>2022,962</td><td>-0,4</td></tr>
<tr><td></td><td></td><td></td><td></td><td></td><td></td><td></td><td>t</td><td>2</td><td>15,31</td><td>15,33</td><td><0.1%</td><td>15,31</td><td>97,30</td><td>5,20E+06</td><td>153</td><td>(R)Q\Q|C/C\Q/Q/L/R(Q)</td><td>560,7533</td><td>1120,499</td><td>0,6</td></tr>
<tr><td></td><td></td><td></td><td></td><td></td><td></td><td></td><td>c</td><td>2</td><td>16,26</td><td>17,63</td><td><0.1%</td><td>11,92</td><td>88,20</td><td>2,30E+05</td><td>177</td><td>(L)Q S I\L\Q\Q|Q|P Q|S G Q|V|A G L(L)</td><td>841,4485</td><td>1681,887</td><td>1,8</td></tr>
<tr><td></td><td></td><td></td><td></td><td></td><td></td><td></td><td>c</td><td>2</td><td>16,03</td><td>18,87</td><td><0.1%</td><td>12,15</td><td>97,20</td><td>1,60E+06</td><td>177</td><td>(L)Q S I\L\Q\Q\Q|P Q\S G Q V|A\G|L\L(A)</td><td>897,9877</td><td>1794,971</td><td>-1,5</td></tr>
<tr><td></td><td></td><td></td><td></td><td></td><td></td><td></td><td>c</td><td>2</td><td>12,19</td><td>13,82</td><td><0.1%</td><td>7,52</td><td>84,90</td><td>3,75E+05</td><td>181</td><td>(L)Q Q\Q|P Q S G Q\V|A|G|L(L)</td><td>620,8191</td><td>1240,628</td><td>2,3</td></tr>
<tr><td></td><td></td><td></td><td></td><td></td><td></td><td></td><td>c</td><td>2</td><td>15,45</td><td>14,97</td><td><0.1%</td><td>10,10</td><td>96,10</td><td>6,17E+06</td><td>181</td><td>(L)Q Q Q|P Q\S G Q\V|A|G|L\L(A)</td><td>677,3632</td><td>1353,712</td><td>5,2</td></tr>
<tr><td></td><td></td><td></td><td></td><td></td><td></td><td></td><td>c</td><td>2</td><td>11,77</td><td>13,47</td><td><0.1%</td><td>2,13</td><td>82,50</td><td>1,33E+06</td><td>193</td><td>(L)L A\A\Q\I\A\Q|Q|L(T)</td><td>478,2849</td><td>955,557</td><td>5,7</td></tr>
<tr><td></td><td></td><td></td><td></td><td></td><td></td><td></td><td>c</td><td>2</td><td>16,63</td><td>14,82</td><td><0.1%</td><td>13,58</td><td>98,00</td><td>8,31E+05</td><td>202</td><td>(L)T A M C G L|Q|Q|P T|P/C|P Y(A)</td><td>812,3558</td><td>1623,696</td><td>4,9</td></tr>
<tr><td></td><td></td><td></td><td></td><td></td><td></td><td></td><td>c</td><td>2</td><td>17,66</td><td>14,73</td><td><0.1%</td><td>6,77</td><td>94,10</td><td>1,21E+07</td><td>216</td><td>(Y)A/A|A|G|G/V/P/H(-)</td><td>340,1775</td><td>679,352</td><td>-6,6</td></tr>
<tr><td>4A</td><td>P08031.1</td><td>16 (34)</td><td>60%</td><td>217,07</td><td>1,13E+08</td><td>94%</td><td>c</td><td>2</td><td>14,72</td><td>12,95</td><td><0.1%</td><td>10,30</td><td>89,60</td><td>2,01E+07</td><td>45</td><td>(F)Y L|P P Q Q|Q|P Q|P/W(Q)</td><td>691,3532</td><td>1381,690</td><td>6,7</td></tr>
<tr><td></td><td></td><td></td><td></td><td></td><td></td><td></td><td>c</td><td>2</td><td>18,07</td><td>17,71</td><td><0.1%</td><td>18,07</td><td>96,60</td><td>4,28E+06</td><td>56</td><td>(W)Q Y P T\Q|P/P Q\L|S|P/C Q/Q F(G)</td><td>909,9251</td><td>1818,848</td><td>-2,8</td></tr>
<tr><td></td><td></td><td></td><td></td><td></td><td></td><td></td><td>c</td><td>2</td><td>17,35</td><td>15,14</td><td><0.1%</td><td>13,15</td><td>95,30</td><td>4,35E+06</td><td>71</td><td>(F)G S C G\V|G S|V|G/S|P F(L)</td><td>555,7497</td><td>1110,488</td><td>3,3</td></tr>
<tr><td></td><td></td><td></td><td></td><td></td><td></td><td></td><td>c</td><td>2</td><td>11,6</td><td>13,06</td><td><0.1%</td><td>4,44</td><td>87,70</td><td>3,84E+06</td><td>90</td><td>(F)L R\H\Q C S|P A\A|T|P\Y(G)</td><td>700,8417</td><td>1400,674</td><td>1,6</td></tr>
<tr><td></td><td></td><td></td><td></td><td></td><td></td><td></td><td>c</td><td>2</td><td>11,84</td><td>12,10</td><td><0.1%</td><td>5,92</td><td>95,80</td><td>3,09E+06</td><td>91</td><td>(L)R H\Q C S|P A\A|T|P\Y(G)</td><td>644,2968</td><td>1287,590</td><td>-2,8</td></tr>
<tr><td></td><td></td><td></td><td></td><td></td><td></td><td></td><td>c</td><td>1</td><td>12,26</td><td>12,71</td><td><0.1%</td><td>12,26</td><td>93,20</td><td>3,42E+06</td><td>102</td><td>(Y)G S/P/Q|C\Q\A\L(Q)</td><td>860,3923</td><td>860,393</td><td>-0,9</td></tr>
<tr><td></td><td></td><td></td><td></td><td></td><td></td><td></td><td>t</td><td>2</td><td>15,73</td><td>12,48</td><td><0.1%</td><td>7,50</td><td>78,60</td><td>9,38E+06</td><td>119</td><td>(R)Q/V|E|P/L/H/R(Y)</td><td>439,7476</td><td>878,484</td><td>4,2</td></tr>
<tr><td></td><td></td><td></td><td></td><td></td><td></td><td></td><td>c</td><td>2</td><td>13,36</td><td>12,11</td><td><0.1%</td><td>5,93</td><td>85,40</td><td>8,87E+04</td><td>131</td><td>(Y)G V\V\L/Q/S|F|L(Q)</td><td>431,7544</td><td>862,503</td><td>-2,0</td></tr>
<tr><td></td><td></td><td></td><td></td><td></td><td></td><td></td><td>c</td><td>2</td><td>10,51</td><td>11,65</td><td><0.1%</td><td>10,51</td><td>75,30</td><td>7,96E+05</td><td>135</td><td>(L)Q S F\L\Q\Q|Q|P Q\G E L(A)</td><td>701,8508</td><td>1402,696</td><td>-1,3</td></tr>
<tr><td></td><td></td><td></td><td></td><td></td><td></td><td></td><td>c</td><td>2</td><td>12,79</td><td>13,51</td><td><0.1%</td><td>12,79</td><td>91,10</td><td>1,74E+06</td><td>138</td><td>(F)L Q\Q\Q/P Q|G\E|L(A)</td><td>520,7746</td><td>1040,537</td><td>4,6</td></tr>
<tr><td></td><td></td><td></td><td></td><td></td><td></td><td></td><td>c</td><td>2</td><td>18,75</td><td>17,93</td><td><0.1%</td><td>18,75</td><td>100,00</td><td>2,69E+06</td><td>138</td><td>(F)L Q\Q\Q|P Q\G\E|L|A|A|L(M)</td><td>648,3533</td><td>1295,695</td><td>3,0</td></tr>
<tr><td></td><td></td><td></td><td></td><td></td><td></td><td></td><td>c</td><td>2</td><td>10,36</td><td>11,48</td><td><0.1%</td><td>5,06</td><td>87,50</td><td>3,94E+05</td><td>139</td><td>(L)Q Q Q\P Q G\E\L\A|A|L(M)</td><td>591,8056</td><td>1182,611</td><td>-6,3</td></tr>
<tr><td></td><td></td><td></td><td></td><td></td><td></td><td></td><td>c</td><td>2</td><td>14</td><td>15,74</td><td><0.1%</td><td>6,57</td><td>89,90</td><td>1,84E+05</td><td>147</td><td>(L)A A L m\A\A\Q|V\A|Q|Q\L(T)</td><td>615,8250</td><td>1214,656</td><td>-6,8</td></tr>
<tr><td></td><td></td><td></td><td></td><td></td><td></td><td></td><td>c</td><td>2</td><td>11,29</td><td>13,11</td><td><0.1%</td><td>3,71</td><td>76,90</td><td>1,80E+06</td><td>150</td><td>(L)m A\A\Q\V|A|Q|Q|L(T)</td><td>488,2500</td><td>959,498</td><td>-0,1</td></tr>
<tr><td></td><td></td><td></td><td></td><td></td><td></td><td></td><td>c</td><td>2</td><td>14,56</td><td>16,77</td><td><0.1%</td><td>14,56</td><td>90,50</td><td>1,60E+05</td><td>165</td><td>(L)Q L Q\Q|P G P C|P C N A\A|A|G\G\V Y(Y)</td><td>944,4268</td><td>1887,848</td><td>-0,7</td></tr>
<tr><td></td><td></td><td></td><td></td><td></td><td></td><td></td><td>c</td><td>2</td><td>9,88</td><td>16,24</td><td><0.1%</td><td>9,88</td><td>83,20</td><td>4,69E+04</td><td>167</td><td>(L)Q Q\P G\P C\P C\N\A\A\A\G\G\V/Y(Y)</td><td>823,8532</td><td>1646,705</td><td>-3,6</td></tr>
<tr><td>4B</td><td>P08031.1</td><td>20 (41)</td><td>61%</td><td>302,29</td><td>3,11E+08</td><td>97%</td><td>c</td><td>2</td><td>17,07</td><td>14,32</td><td><0.1%</td><td>10,80</td><td>95,40</td><td>3,65E+06</td><td>33</td><td>(F)H L|P P P F\Y|m|P|P|P F(Y)</td><td>728,3590</td><td>1439,718</td><td>-1,5</td></tr>
<tr><td></td><td></td><td></td><td></td><td></td><td></td><td></td><td>c</td><td>2</td><td>13,04</td><td>13,62</td><td><0.1%</td><td>7,13</td><td>87,00</td><td>6,80E+05</td><td>40</td><td>(Y)M P P P F Y L|P P Q\Q|Q|P Q|P/W(Q)</td><td>975,9806</td><td>1950,957</td><td>-1,6</td></tr>
<tr><td></td><td></td><td></td><td></td><td></td><td></td><td></td><td>c</td><td>2</td><td>15,18</td><td>13,87</td><td><0.1%</td><td>12,18</td><td>87,50</td><td>3,82E+07</td><td>45</td><td>(F)Y L|P P Q\Q|Q|P Q|P/W(Q)</td><td>691,3433</td><td>1381,690</td><td>-7,7</td></tr>
<tr><td></td><td></td><td></td><td></td><td></td><td></td><td></td><td>c</td><td>2</td><td>15,6</td><td>13,06</td><td><0.1%</td><td>10,09</td><td>96,00</td><td>8,42E+05</td><td>46</td><td>(Y)L P P Q Q|Q|P\Q|P/W(Q)</td><td>609,8181</td><td>1218,627</td><td>1,9</td></tr>
<tr><td></td><td></td><td></td><td></td><td></td><td></td><td></td><td>c</td><td>2</td><td>21,7</td><td>22,21</td><td><0.1%</td><td>21,70</td><td>100,00</td><td>8,99E+05</td><td>56</td><td>(W)Q Y P T\Q|P P Q\L|S|P C Q Q F|G\S C/G V|G S/V G/S/P F(L)</td><td>1455,6599</td><td>2910,318</td><td>-2,1</td></tr>
<tr><td></td><td></td><td></td><td></td><td></td><td></td><td></td><td>c</td><td>1</td><td>16,65</td><td>17,21</td><td><0.1%</td><td>12,56</td><td>91,20</td><td>1,38E+07</td><td>71</td><td>(F)G S/C/G/V|G/S|V\G\S|P F(L)</td><td>1110,4802</td><td>1110,488</td><td>-7,4</td></tr>
<tr><td></td><td></td><td></td><td></td><td></td><td></td><td></td><td>c</td><td>2</td><td>16,37</td><td>16,38</td><td><0.1%</td><td>16,37</td><td>91,20</td><td>1,19E+06</td><td>71</td><td>(F)G S C G V|G S V|G S|P F/L|G Q/C V|E F(L)</td><td>972,4254</td><td>1943,863</td><td>-9,8</td></tr>
<tr><td></td><td></td><td></td><td></td><td></td><td></td><td></td><td>t</td><td>2</td><td>15,15</td><td>12,63</td><td><0.1%</td><td>6,96</td><td>80,20</td><td>3,05E+07</td><td>119</td><td>(R)Q/V|E|P/L/H/R(Y)</td><td>439,7490</td><td>878,484</td><td>7,3</td></tr>
<tr><td></td><td></td><td></td><td></td><td></td><td></td><td></td><td>c</td><td>2</td><td>13,52</td><td>12,93</td><td><0.1%</td><td>10,52</td><td>85,40</td><td>8,82E+05</td><td>135</td><td>(L)Q S F L\Q|Q|Q|P Q\G\E L(A)</td><td>701,8453</td><td>1402,696</td><td>-9,1</td></tr>
<tr><td></td><td></td><td></td><td></td><td></td><td></td><td></td><td>c</td><td>2</td><td>14,04</td><td>13,98</td><td><0.1%</td><td>14,04</td><td>91,70</td><td>2,74E+06</td><td>138</td><td>(F)L Q\Q\Q/P Q|G\E|L(A)</td><td>520,7744</td><td>1040,537</td><td>4,3</td></tr>
<tr><td></td><td></td><td></td><td></td><td></td><td></td><td></td><td>c</td><td>2</td><td>18,75</td><td>16,73</td><td><0.1%</td><td>12,94</td><td>100,00</td><td>5,52E+06</td><td>138</td><td>(F)L Q\Q\Q|P Q\G E|L|A|A|L(M)</td><td>648,3478</td><td>1295,695</td><td>-5,5</td></tr>
<tr><td></td><td></td><td></td><td></td><td></td><td></td><td></td><td>c</td><td>2</td><td>8,52</td><td>11,65</td><td><0.1%</td><td>4,73</td><td>79,20</td><td>5,79E+05</td><td>139</td><td>(L)Q Q Q\P Q\G\E\L\A|A|L(M)</td><td>591,8035</td><td>1182,611</td><td>-9,8</td></tr>
<tr><td></td><td></td><td></td><td></td><td></td><td></td><td></td><td>c</td><td>2</td><td>18,68</td><td>20,92</td><td><0.1%</td><td>18,68</td><td>87,90</td><td>6,15E+04</td><td>139</td><td>(L)Q Q\Q\P Q G E L\A|A/L|m/A A|Q V|A|Q/Q L(T)</td><td>1070,0442</td><td>2123,091</td><td>-2,4</td></tr>
<tr><td></td><td></td><td></td><td></td><td></td><td></td><td></td><td>c</td><td>2</td><td>15,34</td><td>17,49</td><td><0.1%</td><td>10,04</td><td>88,00</td><td>4,75E+05</td><td>147</td><td>(L)A A L m\A\A|Q|V|A\Q|Q\L(T)</td><td>615,8238</td><td>1214,656</td><td>-8,7</td></tr>
<tr><td></td><td></td><td></td><td></td><td></td><td></td><td></td><td>c</td><td>2</td><td>13,38</td><td>13,84</td><td><0.1%</td><td>3,96</td><td>84,00</td><td>5,21E+06</td><td>150</td><td>(L)m A\A\Q\V|A|Q|Q|L(T)</td><td>488,2525</td><td>959,498</td><td>5,1</td></tr>
<tr><td></td><td></td><td></td><td></td><td></td><td></td><td></td><td>c</td><td>2</td><td>16,94</td><td>20,15</td><td><0.1%</td><td>16,94</td><td>88,70</td><td>7,89E+04</td><td>150</td><td>(L)m A A\Q\V|A/Q|Q|L|T|A/m|C G L(Q)</td><td>812,8705</td><td>1592,759</td><td>-9,5</td></tr>
<tr><td></td><td></td><td></td><td></td><td></td><td></td><td></td><td>c</td><td>2</td><td>12,5</td><td>14,49</td><td><0.1%</td><td>12,50</td><td>75,00</td><td>1,01E+06</td><td>165</td><td>(L)Q L Q\Q|P G P C|P C N A\A|A|G G\V Y(Y)</td><td>944,4231</td><td>1887,848</td><td>-4,6</td></tr>
<tr><td></td><td></td><td></td><td></td><td></td><td></td><td></td><td>c</td><td>2</td><td>13,58</td><td>14,87</td><td><0.1%</td><td>13,58</td><td>87,50</td><td>1,95E+06</td><td>165</td><td>(L)Q L Q\Q|P G P C|P C N A A|A\G G\V|Y Y(-)</td><td>1025,9511</td><td>2050,911</td><td>-7,8</td></tr>
<tr><td></td><td></td><td></td><td></td><td></td><td></td><td></td><td>c</td><td>2</td><td>14,69</td><td>17,69</td><td><0.1%</td><td>14,69</td><td>90,30</td><td>3,18E+05</td><td>167</td><td>(L)Q Q\P G\P C|P C N A\A\A|G\G|V|Y(Y)</td><td>823,8569</td><td>1646,705</td><td>0,9</td></tr>
<tr><td></td><td></td><td></td><td></td><td></td><td></td><td></td><td>c</td><td>1</td><td>11,59</td><td>11,58</td><td><0.1%</td><td>6,53</td><td>80,50</td><td>2,36E+07</td><td>202</td><td>(L)T/A|M|C\G\L(Q)</td><td>652,2796</td><td>652,279</td><td>0,5</td></tr>
</table>
